# Transcriptomics uncovers substantial variability associated with alterations in manufacturing processes of macrophage cell therapy products

**DOI:** 10.1038/s41598-020-70967-2

**Published:** 2020-08-20

**Authors:** Olga L. Gurvich, Katja A. Puttonen, Aubrey Bailey, Anssi Kailaanmäki, Vita Skirdenko, Minna Sivonen, Sanna Pietikäinen, Nigel R. Parker, Seppo Ylä-Herttuala, Tuija Kekarainen

**Affiliations:** 1 Kuopio Center for Gene and Cell Therapy, Microkatu 1S, 70210 Kuopio, Finland; 2 A.I. Virtanen Institute, University of Eastern Finland, 70211 Kuopio, Finland

**Keywords:** Cell biology, Molecular biology

## Abstract

Gene expression plasticity is central for macrophages’ timely responses to cues from the microenvironment permitting phenotypic adaptation from pro-inflammatory (M1) to wound healing and tissue-regenerative (M2, with several subclasses). Regulatory macrophages are a distinct macrophage type, possessing immunoregulatory, anti-inflammatory, and angiogenic properties. Due to these features, regulatory macrophages are considered as a potential cell therapy product to treat clinical conditions, e.g., non-healing diabetic foot ulcers. In this study we characterized two differently manufactured clinically relevant regulatory macrophages, programmable cells of monocytic origin and comparator macrophages (M1, M2a and M0) using flow-cytometry, RT-qPCR, phagocytosis and secretome measurements, and RNA-Seq. We demonstrate that conventional phenotyping had a limited potential to discriminate different types of macrophages which was ameliorated when global transcriptome characterization by RNA-Seq was employed. Using this approach we confirmed that macrophage manufacturing processes can result in a highly reproducible cell phenotype. At the same time, minor changes introduced in manufacturing resulted in phenotypically and functionally distinct regulatory macrophage types. Additionally, we have identified a novel constellation of process specific biomarkers, which will support further clinical product development.

## Introduction

Macrophage-based cell therapies have already reached the clinical trial phase, however, understanding of the different macrophage types is still limited. We are primarily interested in regulatory macrophages (Mreg and Mreg_UKR), which are currently tested in Phase I/II clinical trial in kidney transplantations^1–3^. Pre-clinical data suggest their role in modulating immune responses, preventing immunopathologies associated with prolonged inflammation, and restricting fibrosis^1,4–7^.

Macrophages are plastic immune cells present in a variety of tissues throughout the body. Their activation and polarization are highly dependent on microenvironment, which facilitates the acquisition of the macrophage phenotype. Differentially polarized macrophages have their corresponding roles in immune responses, tissue homeostasis and regeneration, and in wound healing progression^8,9^. Following a breach of the host's epithelial barrier, macrophages sense the presence of foreign matter such as bacterial lipopolysaccharides (LPS), free heme, or DNA. The induced cascade of transcriptional responses, augmented by interferon gamma (IFN-ɣ) released from T helper 1 (T_H_1) cells, promotes acquisition of the M1 pro-inflammatory phenotype^10^. Macrophages polarized to the M1 phenotype release a variety of inflammatory cytokines as well as antimicrobial peptides and proteins. Whereas M1 polarization leads to pro-inflammatory response, M2 macrophages dampen inflammation, facilitate wound closure and promote tissue remodelling^11,12^. M2 macrophages exhibit a variety of sub-phenotypes evoked by a panoply of stimulating signals. For example, M2a-phenotype is acquired in the response to IL-4 and IL-13 cytokines and this macrophage type assists wound healing by promoting extracellular matrix formation and angiogenesis.

Macrophage plasticity and their central role in immune response and wound healing, makes them an attractive candidate cell therapy for the amelioration of inflammatory pathologies, with few being tested in clinics so far. In one study, injections of donor-derived, osmotically-activated macrophages showed better efficacy in healing pressure ulcers in elderly patients when compared to standard wound care^13^. In another study, autologous skin-activated macrophages were evaluated for the treatment of spinal cord injuries (SCI)^14^. While the phase I clinical trial showed certain improvement in motor and sensory function in some subjects, the phase II trial failed to demonstrate significant difference between the treatment and control groups. Nevertheless, both trials demonstrated safety of the macrophage therapy in SCI^15^. A phase I-II clinical trial^1,3^ demonstrated that administration of donor-derived, ex vivo differentiated Mreg and Mreg_UKR to kidney transplant recipients prior to transplantation, promoted transplant tolerance. Another ongoing trial aims to prove the efficacy of autologous, ex vivo differentiated macrophages for the treatment of liver cirrhosis^16,17^. In a relevant, but less directly-related study, another monocyte derivative, programmable cells of monocytic origin (PCMO)^18^ were clinically tested to treat critical limb ischemia (CLI) by injecting into the anteromedial and posterior muscle compartments of the lower leg of 4 patients. Improved angiogenesis and wound healing were reported by authors^19^.

Accurate characterization is pivotal to standardizing cell therapy production parameters and to ensuring consistency. The current clinically used macrophage products are manufactured by a variety of methods and the product characterization has been mainly limited to the cell surface protein determination by flow cytometry^1,16^ or measuring the expression of released cytokines^16^. Meanwhile, there is a clear need for objective, global characterization, which can be acquired *e.g.* by RNA-Seq analysis. Transcriptome studies on human macrophages using RNA-Seq are still limited, the majority of which have been performed using microarrays and/or to answer specialized questions^20^. Earlier transcriptome studies on human M1- and M2a-polarized macrophages had discovered novel sets of molecules and signatures^9,21–26^. Subsequent cumulative re-analysis of the published data yielded additional candidate genes ostensibly involved in macrophage polarization^27,28^. One transcriptome study of macrophages used 29 different stimuli to mimic the cue sets a cell might encounter in vivo purported to confirm the “spectrum” model of macrophage activation^29^. Another study compared both sequencing and microarray approaches and concluded that RNA-Seq data revealed greater differences between M1-like and M2-like cells than the data obtained from microarrays^23^.

Here we report a comprehensive study of differently manufactured macrophages, with the specific emphasis on regulatory macrophages, namely Mreg^1,30^ and Mreg_UKR^31^. These regulatory macrophage protocols differ by two key points: choice of culture vessel and frequency of medium replenishment throughout macrophage maturation. Presented here, these seemingly minor differences result in two distinct products with unique transcriptional patterns. In parallel, non-polarized M0, proinflammatory M1, and alternatively activated M2a, as well as PCMO-like cells, were produced and compared. Using this unbiased characterization, a novel constellation of process-specific biomarkers for each cell type was identified. These data will support the development of future regulatory macrophage cell therapy products and guide the assessment of critical quality attributes relevant to the mode of action and safety.

## Materials and methods

### Monocyte enrichment

Highly pure (98 ± 1%) and viable (98 ± 2%) monocytes from a leukapheresis product were obtained utilizing LP14 Process in CliniMACS Prodigy (Miltenyi Biotec GmbH) according to manufacturer’s instructions.

### Macrophage differentiation methods

**Figure 1:**
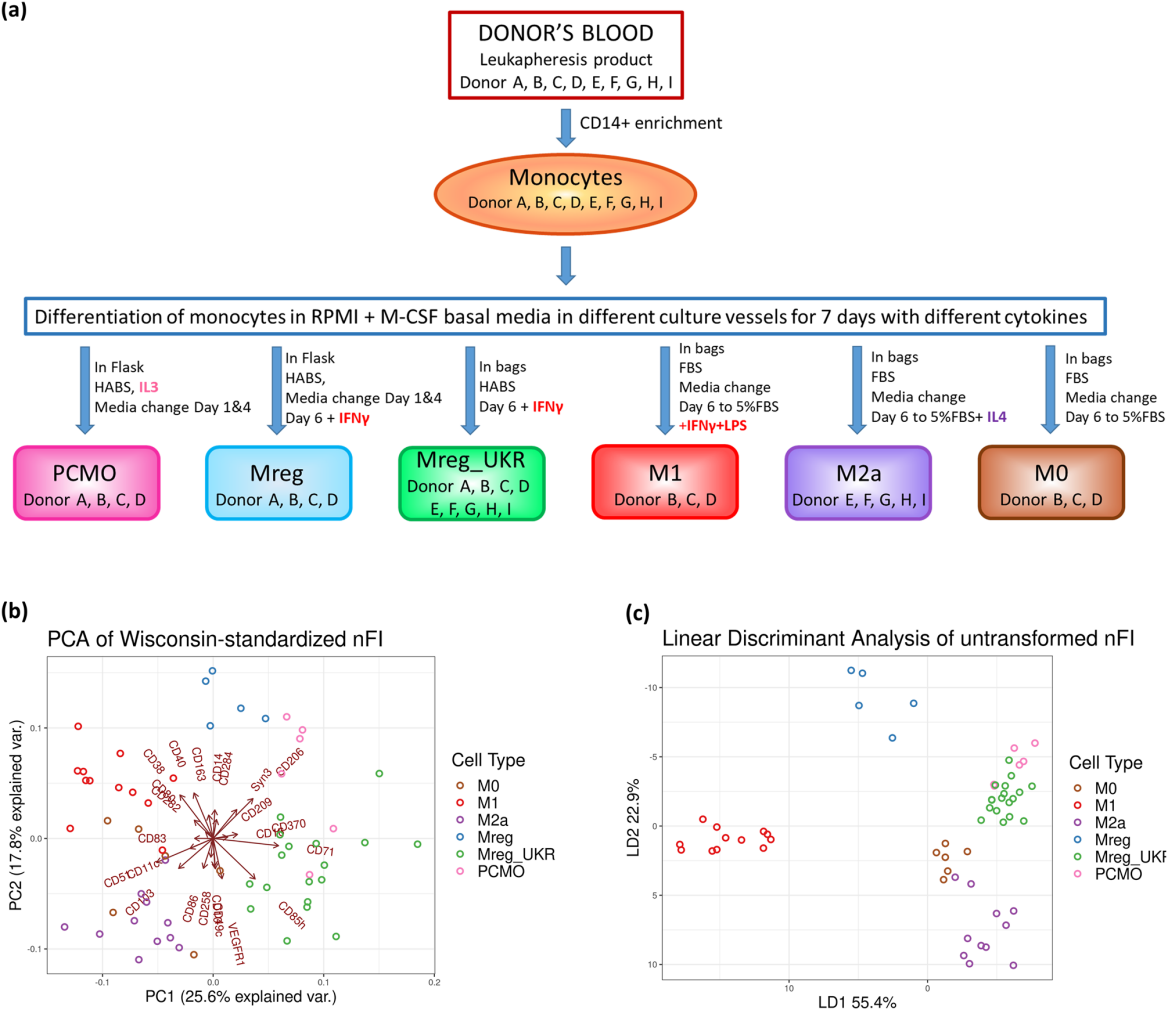
Produced macrophages and their phenotype. (**a**) Macrophage manufacturing procedures; (**b**) PCA of Wisconsin-standardized nFI of 23 macrophage-associated extracellular markers. The direction and the magnitude of the vector arrows denotes the relative strength of each marker within each sample and informs its placement within the figure; (**c**) LDA ordination of samples by cell types, using all markers. Axes are labeled with percent variability explained by each discriminant.

As schematically presented in Fig. 1a, purified monocytes were differentiated into M0, M1, M2a, Mreg type-of-cells or PCMO-like cells according to the published protocols with minor modifications.

*Mreg_UKR cells* were manufactured according to published protocol^31^ in gas-permeable MACS GMP differentiation bags (Miltenyi Biotec). Monocytes were seeded at 1 × 10^6^ cells/ml in RPMI 1,640 (Lonza) medium supplemented with 10% human AB serum (Sigma), 2 mM GlutaMAX (Invitrogen, 100 U/ml penicillin, 100 µg/ml streptomycin (Invitrogen), and 25 ng/ml recombinant human M-CSF (R&D Systems). Cells were cultured in a humidified atmosphere at 37 °C, with 5% CO_2_ for 7 days. On day 6, IFN-ɣ (Merck) was added at a final concentration of 25 ng/ml.

*Mreg cells* were differentiated from purified monocytes for 7 days similarly to Mreg_UKR cells, but in Cell + flasks (Sarstedt)^1,30^ and with the following modifications: (1) medium was changed twice during the process (day 1 and 4); and (2) cells were harvested by scraping.

*PCMO-like cells* were cultured and harvested similarly to Mreg cells, but in the presence of 0.4 ng/ml of recombinant human IL-3 (Miltenyi Biotech) and without addition of IFN-ɣ on day 6. This production method deviates from the one originally reported for PCMO^19,32^ to better fit the scheme and timelines of the manufacturing of other cells. Because of these changes in manufacturing, we call these cells PCMO-like throughout the text.

*M0, M1 and M2a cells *were produced according to protocols given in Hutchinson et al.^30^ in the gas-permeable MACS GMP bags. Cells were differentiated for 6 days in RPMI 1,640/GlutaMAX/antibiotics (same as for Mreg_UKR), containing 5 ng/ml M-CSF and 20% foetal bovine serum (FBS; Gibco). On day 6 medium was exchanged, serum concentration reduced to 5% FBS and M-CSF increased to 25 ng/ml. M1 cells were polarized with 100 ng/ml of lipopolysaccharides (LPS, Sigma-Aldrich) and 25 ng/ml IFN-γ, while M2a cells were polarized with 20 ng/ml IL-4 (R&D Systems). M0 cells did not receive any additional cytokines. Cells were harvested on day 7.

### Flow cytometric characterization

Flow cytometry data was acquired by CytoFLEX S (Beckman Coulter) and analyzed with FCS Express 6 Flow Research Edition (DeNovo Software). The percentage of CD14 + monocytes and CD3 + lymphocytes from all CD45 + white blood cells were determined from the initial leukapheresis product and after monocyte enrichment. The cells were blocked with FcR Blocking Reagent (Miltenyi Biotec) and stained with CD45-PE, CD3-BV421 and CD14-APC antibodies and 7-AAD to assess viability. For all differentiated cells a total of 23 surface markers (CD10, CD11c, CD14, CD16, CD38, CD40, CD49c, CD51, CD71, CD80, CD83, CD85h, CD86, CD103, CD163, CD206, CD209, CD258, CD282, CD284, CD370, Syn3, VEGFR1) were determined as detailed in Supplementary Table S1. Extracellular markers were analyzed from live macrophages, defined as CD45-positive, viability dye (SYTOX Green) negative events. Normalized median fluorescence intensity (nMFI) was calculated by subtracting the nMFI for the CD45-only stain from each measurement. The normalized nMFIs were ordinated using linear discriminant analysis (LDA) using the MASS v7.3.51.4^33^ package in R. For indoleamine 2,3-dioxygenase (IDO) intracellular staining cells were fixed and permeabilized using Intracellular Fixation and Permeabilization Buffer Set (eBioscience). The IDO signal was analyzed from CD33-positive, eFluor 660 Fixable Viability Dye-negative events.

### Cytokine and growth factor secretion profiles

For secretome analysis, culture medium supernatants were collected during harvest. All the samples were stored at − 80 °C until analysis with Bio-Plex Pro Human Cytokine 27-plex Assay kit (Bio-Rad Laboratories) according to the manufacturer’s instructions using Bio-Plex 200 system (Bio-Rad). Data were analyzed with Bio-Plex Manager software version 4.1.1, and visualized utilizing R. In order to compare multiple assays, median fluorescence intensities (MFI) were first normalized (nFI) by subtracting the background measurement from each analyte. Principle coordinates were calculated in R in order to assess the degree of inter-assay technical variability in comparison with experimental, inter-cell-type variability. Due to the inter-assay variability, a reduced resolution, binned heatmap method was employed. The R package dr4pl v1.1.11^34^ was used to create a combined standard curve from which the binning thresholds were established. Bins were drawn from the upper and lower asymptotes, as well as the calculated midpoint (EC50).

### Gene expression characterization by RT-qPCR

Total RNA was extracted from cells using RNeasy Protect Cell Mini Kit (Qiagen) with QIAshredder homogenizers (Qiagen). RNA was treated with DNase I (PrimerDesign or Invitrogen) according to manufacturer’s instruction, samples’ concentration was adjusted to 50 ng/µl and verified on Qubit Fluorometer (Invitrogen) using Qubit RNA HS Assay Kit (Invitrogen). Expression of *DHRS9* and *Ido1* mRNA was measured by RT-qPCR relative to *GAPDH* mRNA endogenous control using TaqPath 1-Step Multiplex Master Mix Kit (Applied Biosystems) and QuantStudio 5 Real-time PCR system (Applied Biosystems) according to manufacturer’s instruction. TaqMan assays were from Applied Biosystems: for Human Ido-1 assay number Hs00984148_m1 (FAM-MGB), for Human DHRS9 Hs00608375_m1 (FAM-MGB) and for Human GAPDH Hs03929097_g1 (VIC-MGB). Each amplification reaction contained 50 ng of RNA and was performed in triplicates. Data was analyzed with QuantStudio Design and Analysis desktop Software (Applied Biosystems). Changes in expression (Rq) were calculated relative to expression of corresponding mRNA in the starting material, i.e. CD14 + monocytes.

### Phagocytosis assay

The flow cytometry-based phagocytosis assay was performed with harvested macrophages, CD14 + monocytes and CD3 + T cells using the pHrodo Green *E. coli* BioParticles Phagocytosis Kit for Flow Cytometry (Invitrogen) according to the manufacturer’s protocol. One million macrophages/monocytes and half a million of T cells were incubated with pHrodo Green *E. coli* BioParticles Conjugate for 1 h at + 37 °C or on ice in order to inhibit particle uptake. Cells were harvested and stained with 7-AAD, CD45-PE/Cy7 (Biolegend), and CD3-BV421 (Biolegend) for T-cell samples. For analysis, a minimum of 4 × 10^4^ live cells (doublet-discriminated CD45-positive, 7-AAD-negative) were acquired and analyzed in the FlowJo software (TreeStar). Phagocytosis was determined as an increase in pHrodo Green fluorescence relative to the one observed with control sample, incubated on ice, and calculated as the percentage of pHrodo Green-positive cells.

### RNA-Seq sample preparation

Enriched monocytes and macrophages were harvested directly into RNAprotect Cell reagent (Qiagen) and frozen at − 80 °C. RNA extraction and sequencing were performed by Eurofins Genomics to a minimum of 30 M reads using Illumina HiSeq 4000 with 2 × 150 bp paired-end sequencing procedure.

### RNA-Seq data analysis

#### Quality control

Quality of the RNA-Seq reads was inspected using MultiQC v1.7^35^.

#### Read alignment and read counts

RNA-Seq reads were aligned the using STAR aligner v2.5.2^36^, against the Ensembl GRCh38 patch 12 release 95 of the human genome. The aligned reads were counted using the Bioconductor SummarizeOverlaps in ‘union’ mode. Reads were tallied from the reverse strand, using the hidden argument ‘preprocess.reads = invertStrand’. Under-sequenced samples were combined post-quantitation, using the DESeq2 collapseReplicates function of DESeq2 R package^39^ v1.24.0. Read counts were normalized using the variance stabilizing transformation (VST).

#### Hierarchical clustering analysis

Ensembl gene VST values from the sequenced samples were clustered by Ward2 clustering using the Pearson correlation coefficient as a distance metric. Trees were resampled to 1,000 bootstrap iterations using pvclust v2.0-0^40^.

#### Differentially expressed genes

The log_2_-fold changes (LFC) were calculated post-shrinkage using apeglm v1.6.0^42^ with an expected LFC threshold of 1. To be considered significant, a gene was required to have s-value ≤ 0.01, |LFC|≥ 1 and base-mean ≥ 5 (LFC1). Additionally, where specified, more stringent criteria, s-value ≤ 0.0001, |LFC|≥ 4 and base-mean ≥ 40 (LFC4) were applied for *e.g.* M1/M2a polarization marker analyses.

#### Visualisations of genes

Volcano plots of differentially expressed genes were created using ggplot2^43^. Gene expression heatmaps display VST-transformed values and are plotted using pheatmap v1.0.12.

#### Enrichment analysis

Gene set enrichment analysis was performed using the gene sets as collected under MSigDB v7.0^44,45^. Enrichment was calculated using fgsea v1.10.1^46^. The Reactome^47^ and the immunologic signatures^48^ datasets were selected for presentation. Enrichments with |NES|> 1 and adjusted p-value < 0.01 were considered significant. The Database for Annotation, Visualization and Integrated Discovery (DAVID 6.8)^49,50^ was also used to identify overrepresented biological terms in KEGG pathways and Gene Ontology^51,52^ set enrichments. Protein–protein interaction networks in specific gene sets were analyzed using STRING^53^.

Further gene set enrichment, networks, predicted activations, and functional analyses were performed using Qiagen Ingenuity Pathway Analysis (IPA)^54^. Functional characterizations were filtered and visualized in R and ggplot2. “Canonical pathway” activation predictions were considered significant if they exhibited a p-value less than 10^–4^.

“Biofunction activation” predictions were considered significant if they exhibited a B-H corrected p-value < 0.05 and a bias-corrected z-score > 2. Predictions that referred to a disease, cancer, abnormality, or injury state were ignored. Results which were flagged for bias had their bias-corrected z-score substituted for the uncorrected value, per vendor documentation.

### Statistical analysis

Statistical analyses for assays other than RNA-Seq were performed using GraphPad Prism 5.04. Results are expressed as mean ± SD. Comparisons between groups were performed using one-way ANOVA with Tukey’s or Dunnett’s post hoc test. P < 0.05 was defined as statistically significant. The relationship between fold-changes in gene expression in RNA-Seq and RT-qPCR analysis was investigated using linear regression.

### Ethics

Peripheral blood monocytes were isolated from healthy donors’ leukapheresis products. Informed consent was obtained from all donors in accordance with the Declaration of Helsinki. The studies were approved by the Research Ethics Committee of the Northern Savo Hospital District (approval number 186/2017).

## Results

### Generation and initial characterization of macrophages

The three potential cell therapy products, Mreg, Mreg_UKR, and PCMO as well as comparator macrophages, M0, M1, and M2a were differentiated from donor-derived monocytes according to published protocols as outlined in Fig. 1a. All products were assessed by flow cytometry for 23 surface markers. Only a few of the screened extracellular proteins were suitable to specifically identify each cell type (Supplementary Figure S1). Therefore, we utilized a principle component analysis (PCA) of Wisconsin-standardized nFI consisting of all the 23 markers, and identified each cell type with better confidence (Fig. 1b).

The most prominent differences between each cell type were observed within the levels of activation-associated, pro-inflammatory markers. In accordance with the subsequent RNA-level findings (see below), CD38 and CD40 were subtly upregulated in Mreg cells, while M1 macrophages displayed markedly elevated levels of CD38, CD40 and CD80 (p < 0.001 for CD38 and CD80, and p < 0.05 for CD40), when compared to other macrophage types (Supplementary Figure S1). However, this limited set of functionally related markers aside, only a few others, e.g. CD71 for Mreg_UKR, offered a reliable way to identify a specific cell type if employed alone. This is not surprising due to the shared starting material, as well as the known plasticity and overlapping functionality of different macrophage phenotypes^27,36,37^. When used in conjunction with one another the full panel of markers provided sufficient information to unsupervised machine learning techniques that linear discriminant analysis (LDA) (Fig. 1c) and Wisconsin-standardized PCA were able to discern between most cell types (Fig. 1b). Only the M0 cell type defied attempts at classification. The resultant ordination shows each cell type cluster arranged radially around a highly variable M0 core, with each component loading vector, indicating the relative importance of each marker in informing the location of each sample in a cell type cluster.

### Unbiased classification of macrophages by RNA-Seq

**Figure 2:**
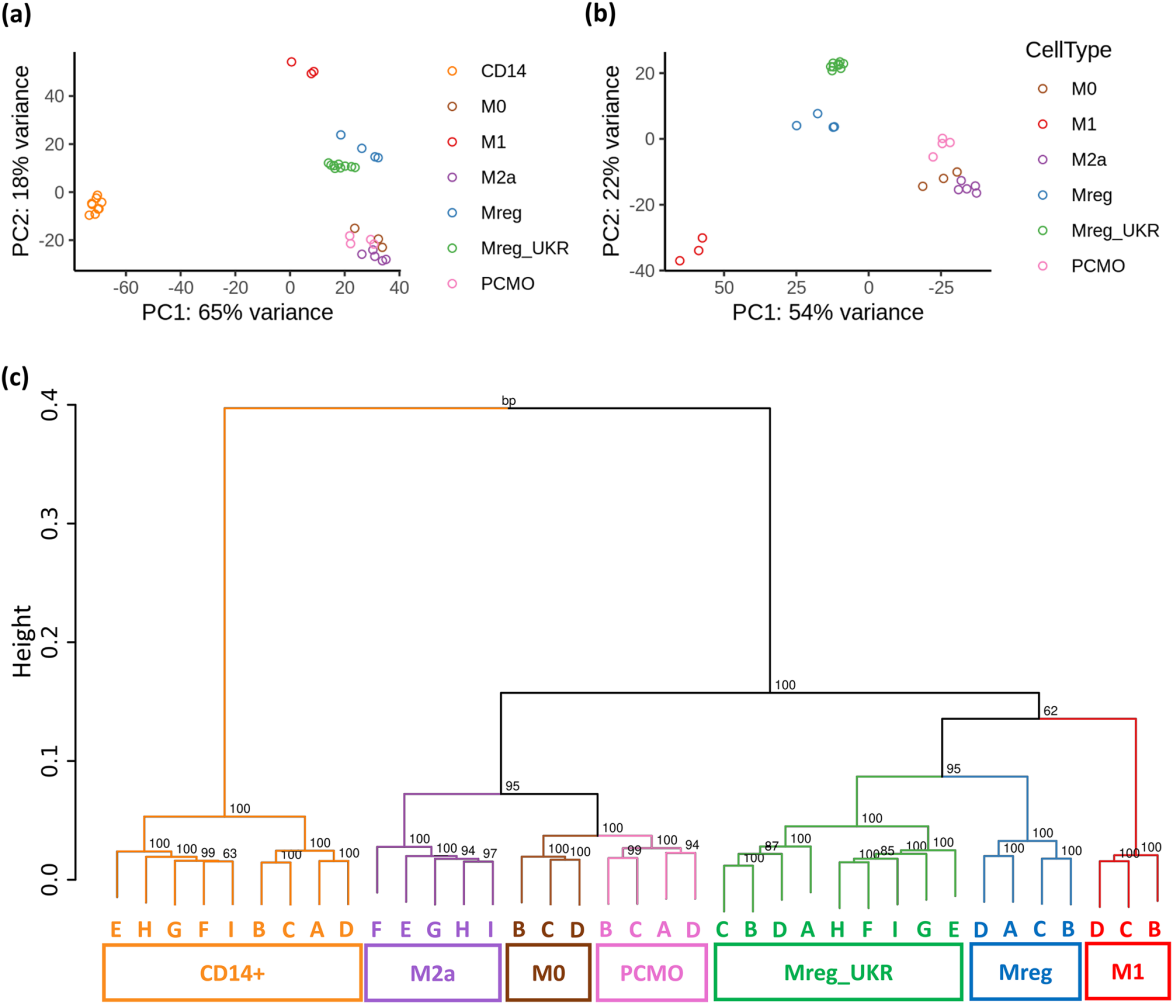
Consistency of macrophage manufacturing procedures. (**a**) PCA analysis of RNA-Seq data from the produced macrophages with and (**b**) excluding CD14 + monocytes. (**c**) Hierarchical clustering dendrogram showing the correlation distances between all RNA-Seq samples. Bootstrap p-values are shown in the nodes and are based on 1,000 replications.

For an in-depth, unbiased characterization of the produced macrophages, the transcriptome of the cells was assessed by RNA-Seq. As expected, all macrophage types were clearly distinct from monocytes by principal component analysis (PCA) and hierarchical clustering of the samples (Fig. 2). The first principal component (PC1 in Fig. 2a) explained 65% of the variance mainly driven by the difference between monocytes and all macrophages that were produced. The PC2 separated the differentiated macrophages and explained 18% of the variance, with M1 (pro-inflammatory) and M2a (wound healing) macrophages showing the furthest separation. Consistent with PCA analysis, the highest number of differentially expressed genes (DEGs), > 5,000, was observed between monocytes and each of the produced macrophages, followed by the M1 vs M2a macrophages with over 3,000 DEGs (Table 1). Together, Mreg_UKR and Mreg formed a common cluster of regulatory macrophages between M1 and M2a. The PCMO-like cells were highly similar to M2a and M0 macrophages and dissimilar to the regulatory macrophages as reflected in PCA and in the low number of DEGs observed between M2a and PCMO-like cells vs non-polarized M0.

**Table 1:** Number of differentially expressed genes between different cell types (s-value < 0.01, absolute log_2_ fold-change > 1).

Comparison name	Differentially expressed genes		
	All genes	Protein-coding upregulated	Protein-coding downregulated
M2a_vs_CD14	6,637	2,645	2074
M1_vs_CD14	6,251	2,608	2030
M0_vs_CD14	6,050	2,431	2087
PCMO_vs_CD14	5,880	2,491	1913
Mreg_UKR_vs_CD14	5,781	2,296	1971
Mreg_vs_CD14	5,763	2,302	1954
M1_vs_M2a	3,886	1558	1,457
PCMO_vs_M1	3,721	1,467	1565
M1_vs_M0	3,281	1,495	1,239
Mreg_UKR_vs_M1	3,201	1,175	1,360
Mreg_UKR_vs_M2a	2,347	851	960
Mreg_vs_M1	2,241	951	913
Mreg_UKR_vs_M0	1904	752	727
Mreg_vs_M2a	1897	708	526
Mreg_UKR_vs_PCMO	1568	525	721
Mreg_UKR_vs_Mreg	1,002	321	486
PCMO_vs_M2a	983	368	282
Mreg_vs_M0	916	519	253
Mreg_vs_PCMO	849	462	270
M2a_vs_M0	629	204	140
PCMO_vs_M0	184	104	52

Exclusion of CD14 + cells in the gene expression PCA plots facilitated a better separation of all the macrophage types (Fig. 2b). The variation between cell types was always greater than the variation between the donors in the same cell type cluster, indicating the robustness and reproducibility of the manufacturing methods (Figs. 1a and 2b). Although the two regulatory macrophage products, Mreg and Mreg_UKR, were most related to each other, they still had clearly distinct transcriptomes with 1,002 DEGs between them. Nevertheless, the differentiating medium and added cytokine (IFN-γ) were identical. The difference between Mreg and Mreg_UKR appeared to be solely driven by the culture vessel and medium changes in the Mreg process. The hard surface of the flasks and medium replenishment made Mreg cells somewhat more M1-like, when compared to Mreg_UKR. Accordingly, in flow cytometry analysis, slightly elevated levels of CD38 and CD40 were noted for Mreg cells (Supplementary Figure S1). Additionally, just as M1 macrophages, Mreg cells secreted chemoattractants MCP-1, MIP-1a and MIP-1b, which play a role in recruiting other cells to sites of inflammation, but not classical inflammatory cytokines like TNF-α, IL-6, or IL-12 nor pro-inflammatory chemokine RANTES, which were secreted only by M1 (Supplementary Figure S2). From regulatory macrophages, Mreg_UKR were more distinct from M1 and M2a macrophages than Mreg as reflected in the total amount of observed DEGs and protein-coding genes (Table 1).

Hierarchical clustering confirmed that all samples, while distinct, were most closely associated with samples of the same cell type (Fig. 2c). M2a, PCMO and M0 formed a separate branch from cells that were produced with the addition of IFN-γ: M1, Mreg and Mreg_UKR. Within the IFN-γ-stimulated branch, regulatory macrophages clustered separately from M1 cells.

Taken together, the consistency of the manufacturing process and the reproducibility of manufactured cells’ phenotypes were ascertained by the hierarchical clustering and PCA ordination.

### Characterization of comparator phenotype macrophages and PCMO-like cells

**Figure 3:**
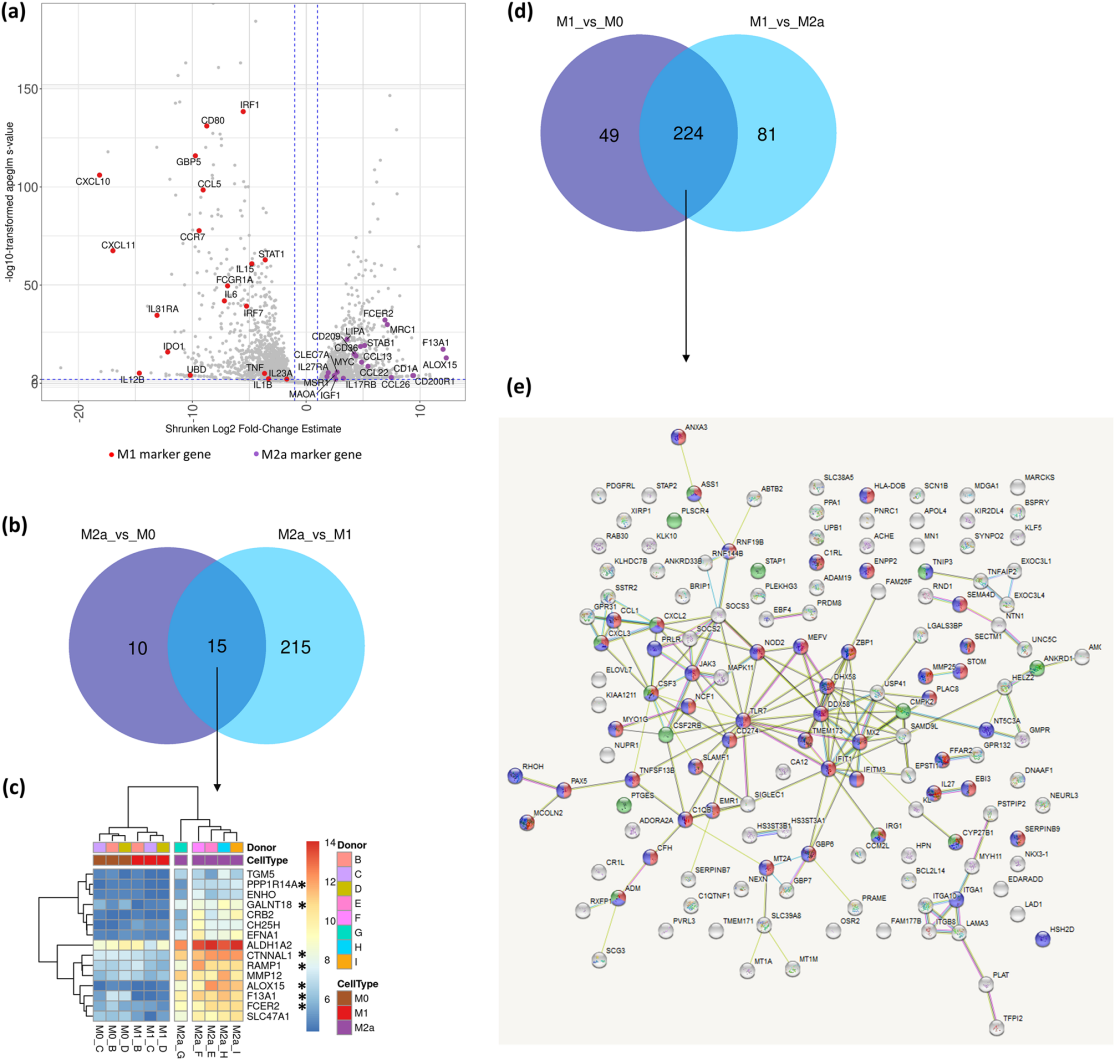
M1/M2a marker genes’ expression. (**a**) Volcano plot of differential gene expression for M2a_vs_M1 comparison between M1 and M2a macrophages, with classical M1 and M2a marker genes indicated. (**b**) Venn diagram analysis to identify genes with highest expression in M2a. Overlap of at least 16-fold upregulated genes (LFC4: log2foldchange > 4, s < 0.0001 and base mean > 40) in M2a_vs_M0 and M2a_vs_M1 comparison defines our M2a marker genes (15) with (**c**) their expression visualized in a heatmap; (**d**) Venn diagram analysis to identify genes with highest expression in M1. Overlap of at least 16-fold upregulated genes (LFC4: log2foldchange > 4, s < 0.0001 and base mean > 40) in M1_vs_M0 and M1_vs_M2a comparison defines our M1 marker genes (224). (**e**) Network analysis using STRING of the gene set overlap identified in (**d**) excluding literature gene set. In red are genes assigned to GO:000695 ~ ”immune response”, in green—GO:0032496 ~ “response to LPS” and in blue—GO:0002376 ~ “immune system process”.

The transcriptomes of M1, M2a and PCMO-like cells were interrogated for expression of their literature-reported phenotype markers. Furthermore, the M1- and M2a-polarized macrophages were compared to each other, and to the non-polarized M0 to identify additional genes induced in response to polarizing agents, IFN-γ/LPS (M1) and IL-4 (M2a). The plethora of previously reported polarization markers was narrowed down to a set of the most widely accepted human marker genes^38^ and complemented with the genes that were reported in previous M1/M2a comparative transcriptome analyses^9,23,25,27^. The full list of examined genes and their expression in produced M1 (162 genes) and M2a (123 genes) macrophages is given in Supplementary Table S2. Overall, the transcriptional profiles of M1 and M2a macrophages conformed to those described in the literature (Fig. 3a and Supplementary Table S2).

To screen for novel M1/M2a polarization markers, we analysed the genes with 16-fold upregulation (LFC4) in M1 and M2a relative to each other and to M0 macrophages. This lead to identification of 224 protein-coding transcripts highly expressed in M1 relative to both M2a and M0 and of 15 highly abundant transcripts in M2a relative to both M1 and M0 (Fig. 3b–e). Of interest were 25 transcripts highly upregulated in M2a in comparison to M0 macrophages (M2a_vs_M0) (Fig. 3b, Table 2). Of these transcripts, 14 were not on our compiled marker list for M2a and therefore were queried against the Gene Ontology’s “biological process” (GOBP) annotations. Four transcripts were associated with wound healing (WNT5B, NFE2, MMP12 and GAS6, GO:0042060), and another one, IRF4, was involved in IL-4/IL-13 signalling (Reactome:R-HSA-6785807). Among the 224 most highly expressed M1 transcripts (Fig. 3d), 79 were previously reported markers (Supplementary Table S2). Analysis of functional interactions between the unreported M1 145 marker genes (Table 2) using STRING revealed a tight gene network centred around TLR7/JAK3/IFIT1 genes which are known activators^39–41^ and regulators^42^ of pro-inflammatory response in macrophages with enrichment for the terms: “immune response” (45 genes to GO:0006955), “response to LPS” (14 genes to GO:0032496) and “immune system process” (51 genes to GO:0002376) (Fig. 3e).

**Table 2:** M1 and M2a markers derived from our data (LFC4).

M2a_vs_M0 (25)	**GALNT18**, GAS6, **SPINT2**, **MAOA**, SLC47A1, SCIMP, D**NASE1L3**, ALDH1A2, **F13A1**, **RAMP1**, **CTNNAL1**, TGM5, MMP12, IRF4, ENHO, **FCER2**, NFE2, RBM11, **PPP1R14A**, WNT5B, EFNA1, **ESPNL**, CH25H, CRB2, **ALOX15**
Marker genes upregulated in M2a when compared to M0 and M1 (15)	TGM5, **RAMP1**, ALDH1A2, CH25H, MMP12, **CTNNAL1**, ENHO, SLC47A1, **FCER2**, EFNA1, **PPP1R14A**, **GALNT18**, CRB2, **F13A1**, **ALOX15**
Marker genes upregulated in M1 when compared to M0 and M2a, excluding literature list genes (145)	RNF144B, DHX58, GMPR, MX2, RAB30, KLHDC7B, SIGLEC1, FAM177B, KLK10, LGALS3BP, EXOC3L1, SAMD9L, SSTR2, CXCL2, NT5C3A, MARCKS, TNFSF13B, MAPK11, STOM, FFAR2, CSF2RB, PSTPIP2, NEXN, MMP25, DDX58, SLC39A8, CYP27B1, NCF1, SEMA4D, CXCL3, TNFAIP2, PRDM8, GPR132, KIAA1211, HELZ2, PPA1, TMEM173, CA12, PNRC1, NUPR1, MT2A, SCG3, SOCS2, ITGA10, ENPP2, USP41, SLFN12L, MDGA1, C1RL, CR1L, EPSTI1, IFIT1, ADM, NOD2, RND1, NECTIN3, RNF19B, ITGA1, HS3ST3B1, STAP2, SERPINB9, JAK3, ADGRE1, SCN1B, TLR7, IFITM3, ELOVL7, KLF5, C1QB, MCOLN2, LAMA3, SOCS3, ABTB2, PRLR, CD274, HS3ST3A1, TNIP3, PLEKHG3, CFH, HLA-DOB, BRIP1, ASS1, ANKRD33B, PTGES, ANKRD1, C1QTNF1, BSPRY, CMPK2, ACHE, ITGB8, CCL1, SLAMF1, STAP1, SECTM1, HPN, PDGFRL, MT1M, MT1A, EBI3, DNAAF1, NTN1, LAD1, MEFV, TFPI2, CCM2L, MYH11, XIRP1, ANXA3, ADORA2A, PLAC8, MYO1G, UPB1, HSH2D, MN1, PLSCR4, APOL4, SLC38A5, KL, NKX3-1, KIR2DL4, PAX5, ZBP1, CSF3, NEURL3, EBF4, RHOH, GPR31, GBP6, SERPINB7, EDARADD, PRAME, IL27, OSR2, RXFP1, PLAT, UNC5C, TMEM171, GBP7, AMOTL2, ADAM19, CALHM6, EXOC3L4, SYNPO2, BCL2L14, ACOD1

In bold are the ones confirmed in previous studies for M2a.

Unlike with M1 and M2a macrophages, we found little resemblance of the manufactured PCMO-like cells to the PCMO described in literature. The PCMO-like cells were produced as described previously for PCMO^19,32^ apart from using a highly purified CD14 + population and culturing for a longer time period (7 instead of 4–6 days) without β-mercaptoethanol. Possibly, because of these modifications, no specific reactivation of pluripotency genes (POU5F1 (OCT4), NANOG and MYC)^18^, nor elevated expression of THY1 (CD90), CSF1R (CD115) and IL3RA (CD123)^43^ were observed when compared to either monocytes or to other produced macrophages (Supplementary Table S3 and Supplementary Figure S3a) at least on the RNA level. Only six genes were upregulated in PCMO-like cells relative to all other macrophage types: GNG2, FAXDC2, FGD5, CLVS1, FAIM2, and LYPD1 (LFC1, Supplementary Figure S3b,c). Although produced PCMO-like cells differed from the literature described PCMO, they present an IL-3 induced macrophage subtype and were kept in the analysis to assist in defining the phenotype of regulatory macrophages.

### Regulatory macrophages show intermediate phenotype between M1 and M2a

M1 or M2a polarization resulted in a massive shift in the global gene transcription pattern when compared to non-activated M0 macrophages. Perturbation involved significantly more genes for M1 (3,281) than for the M2a (621). It appears that the default macrophage differentiation, in M-CSF containing medium^9,36^, is driven more towards an M2a phenotype even without the presence of polarizing cytokines. Therefore, comparing M2a vs M1 macrophages in relation to M0 partially masks drastic differences between M2a and M1 transcriptomes (Fig. 3a).

**Figure 4:**
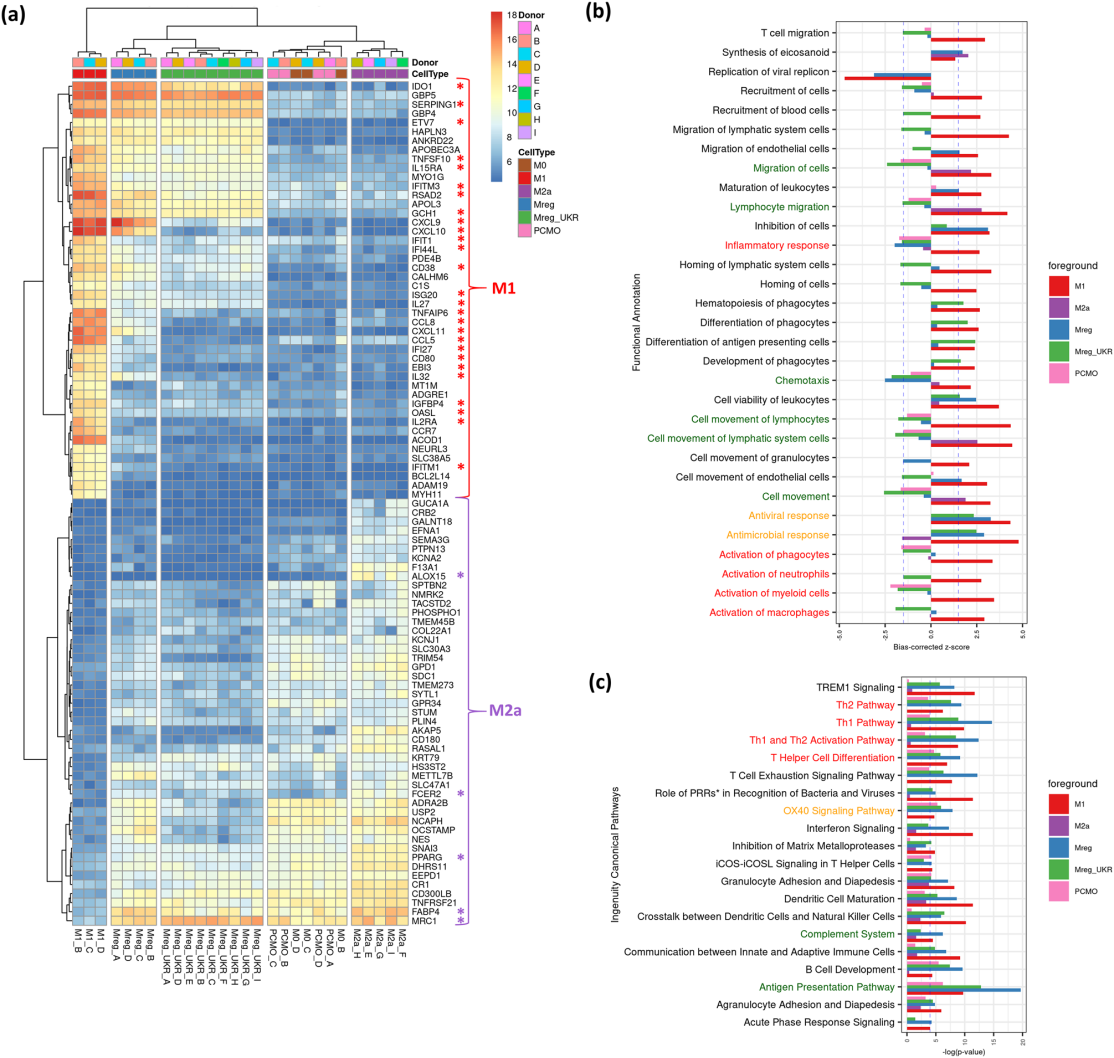
Relation of Mreg, Mreg_UKR, and PCMO-like cells to M1 and M2a polarized macrophages. (**a**) Based on expression of individual M2a/M1 specific genes as defined by 46 most upregulated (M1-specific, basemean > 1,000) and 47 most downregulated (M2a-specific, basemean > 200) in M1_vs_M2a comparison. (**b**) Based on most enriched biofunctions in M1_vs_M0 and M2a_vs_M0 comparisons. (**c**) Based on activated pathways in M1_vs_M0 and M2a_vs_M0 comparisons as determined by IPA. *PRR pattern recognition receptor. The biofunctions and pathways discussed in the text are indicated by different colours.

Because of that, direct a M1_vs_M2a comparison was used to analyse how regulatory macrophages or PCMO-like cells fit into the M1-M2a polarization spectrum on the individual gene expression level. Initially, the 46 most upregulated (M1-specific, basemean > 1,000) and the 47 most downregulated (M2a-specific, basemean > 200) genes were selected and their expression was analysed in regulatory macrophages and PCMO-like cells in relation to M1 and M2a by heatmaps (Fig. 4a). IFN-γ stimulated genes were also induced in Mreg and Mreg_UKR, but to a lesser extent than in M1 macrophages. Nevertheless, some IFN-γ-induced genes, e.g.* CXCL10*, *CXCL11* and *IL32*, expected to be upregulated in Mreg_UKR remained at a similar expression level to M2a and PCMO-like cells. On the other hand Mreg, Mreg_UKR and PCMO-like cells shared increased expression of some M2a-specific genes (Fig. 4a). The expression of mannose receptor *MRC1* (CD206), which is one of the key markers of the M2a phenotype, was higher in Mreg_UKR than in M2a and further supported by flow cytometry (Supplementary Figure S1). While some IL-4-induced genes, *MRC1*, *PPARG* and *FABP4*, were also upregulated in Mreg_UKR, Mreg, and PCMO-like cells, the expression of the others, e.g. *ALOX15* and *FCER2*, was induced only in M2a. The increased expression of the first subset was also noted in unstimulated M0, meaning that the default macrophage maturation likely drives expression of those genes, which are then turned off in the presence of the strong M1 polarization signal such as IFN-γ plus LPS, but not IFN-γ alone.

Next, the DEG data sets were analysed by Ingenuity Pathway Analyser (IPA) to assess changes in the most significant molecular networks, biological functions and canonical pathways between all cell types pairwise. IPA upstream regulator analysis of M2a_vs_M1 and M1_vs_M2a comparisons uncovered key transcriptional regulator networks for M2a and M1 cells, respectively. For M2a the top 3 predicted activated upstream regulators were IL-4, IL-13 and TGFB1, involved in wound healing (Supplementary Table S4), while for M1 the top 5 were LPS, IFN-γ, IFN-α, TNF and IRF7, involved in inflammatory response (Supplementary Table S5). Visualization of the IPA-generated gene networks driven by the identified M2a upstream regulators revealed high expression of the target genes in M2a when compared to M1 macrophages (Supplementary Figure S4a). Similarly, genes in the networks for the identified M1 regulators were highly expressed in M1 when compared to M2a (Supplementary Figure S4b). Both M1 and M2a regulator gene networks were only partially upregulated in Mreg and Mreg_UKR, with a subset of genes in M1 regulator network downregulated in Mreg_UKR (Supplementary Figure S4a,b). The PCMO-like cells showed high expression for a great proportion of the IL-4, IL-13 and TGFB1 regulated genes, but displayed either downregulation or no differential gene expression relative to M2a for M1 regulatory networks. Therefore, major network analysis also supported the intermediate phenotype for Mreg and Mreg_UKR between M1 and M2a, while PCMO-like cells were most similar to M2a.

### Anti-inflammatory and antimicrobial biofunctions are predicted for regulatory macrophages

Additionally, using IPA, we examined the main perturbed pathways and biofunction-activation scores in comparison with M0, used here as a common baseline phenotype. The IPA predicts activated and inhibited biofunctions based on z-score value. We selected activated and inhibited biofunctions with │z-score│ ≥ 2 for M1 and M2a macrophages and investigated predictions for the same biofunctions in Mreg, Mreg_UKR and PCMO-like cells (Fig. 4b). The majority of the predicted activated biofunctions for M1 were related to cell activation, cell migration, inflammatory and antiviral/antimicrobial response. For M2a the main activated biofunctions were related to cell migration. According to IPA analysis Mreg, Mreg_UKR and PCMO-like cells were predicted to behave differently on a number of biofunctions when compared to both M1 and M2a macrophages (Fig. 4b). Specifically, cell movement, migration and chemotaxis z-scores were negative for Mreg, Mreg_UKR and PCMO-like cells. Cell activation z-scores were strongly negative for Mreg_UKR and partially for PCMO-like cells as opposed to M1. Inflammatory response z-scores were negative for Mreg_UKR, Mreg and PCMO-like cells as opposed to M1 and exceeded that of M2a. The z-scores for antiviral and antimicrobial response for both Mreg and Mreg_UKR were in concordance with M1. Thus, based on global transcriptional profile Mreg_UKR, Mreg and PCMO-like cells were predicted to be functionally different from M2a and M1 macrophages. This is specifically intriguing for PCMO-like cells, which based on individual gene expression were similar to M2a and M0, but according to IPA were more anti-inflammatory, with less cell activation and migration properties than M2a and M0. At the same time, Mreg_UKR and Mreg were more anti-inflammatory than the comparator macrophages possessing antimicrobial properties and low cell activation and migration properties.

Functional characterization of DEGs in IPA identified numerous, significantly enriched canonical pathways (FDR-adjusted *P* value ≤ 0.05): 222 pathways for M1_vs_M0, 41 for M2a_vs_M0, 135 for Mreg_vs_M0, 116 for Mreg_UKR_vs_M0, and 43 for PCMO_vs_M0. We selected the most significantly enriched pathways for M1 and M2a with an FDR-adjusted p < 0.00001 and compared the enrichment significance of the same pathways in Mreg, Mreg_UKR, and PCMO-like cells (Fig. 4c). The Antigen Presentation, Complement system, T helper cells differentiation and activation, and OX40 signalling pathways were more significantly enriched in regulatory macrophages than in M1, suggesting that they could be more effective in these functions.

### Regulatory macrophages can be distinguished by genes encoding cell surface proteins

Next, we examined how the transcriptomes of produced Mreg and Mreg_UKR cells correlated with previously published phenotype data for regulatory macrophages. A compiled panel of previously identified regulatory macrophage markers^2,31,38,44^ was used to interrogate gene expression in manufactured cells (Supplementary Table S6 and Supplementary Figure S5a). Hierarchically clustered heatmap analysis for the literature-derived gene set revealed concordant gene expression in Mreg and Mreg_UKR for the majority of these genes. The notable exceptions were *CCL1*^38^, *F13A1*, *XCR1*, *TNFSF18*, *LYVE1* and *CX3CR1*^2^, which were not expressed in our regulatory macrophages. In agreement with the literature, and confirmed by our flow cytometry analysis, expression of mRNA for co-stimulatory molecules CD80 and CD40 was lower in Mreg and Mreg_UKR than in M1. Mreg and Mreg_UKR were generally most positive for expression of immunoglobulin Fc receptor, *FCGR1A*, scavenger receptor *MSR1* and *DHRS9*, which is a recently proposed marker of regulatory macrophages^44^. Nevertheless, with exception for *FCGR1A*, none of these markers exhibited a unique expression pattern that could be used to define regulatory macrophages exclusively and pass a statistical significance test. Indeed, when specifically assessed by RT-qPCR *DHRS9* mRNA expression was found to be induced at similar levels in Mreg_UKR, Mreg and PCMO-like cells relative to the starting monocyte population. The levels of *DHRS9* expression were significantly lower in M2a compared to Mreg_UKR only (p < 0.05) and in M1 when compared to all other cell types (p < 0.001). These results were well correlated between paired measurements in RNA-Seq and RT-qPCR (R^2^ = 0.84) (Supplementary Figure S5b,c).

**Figure 5:**
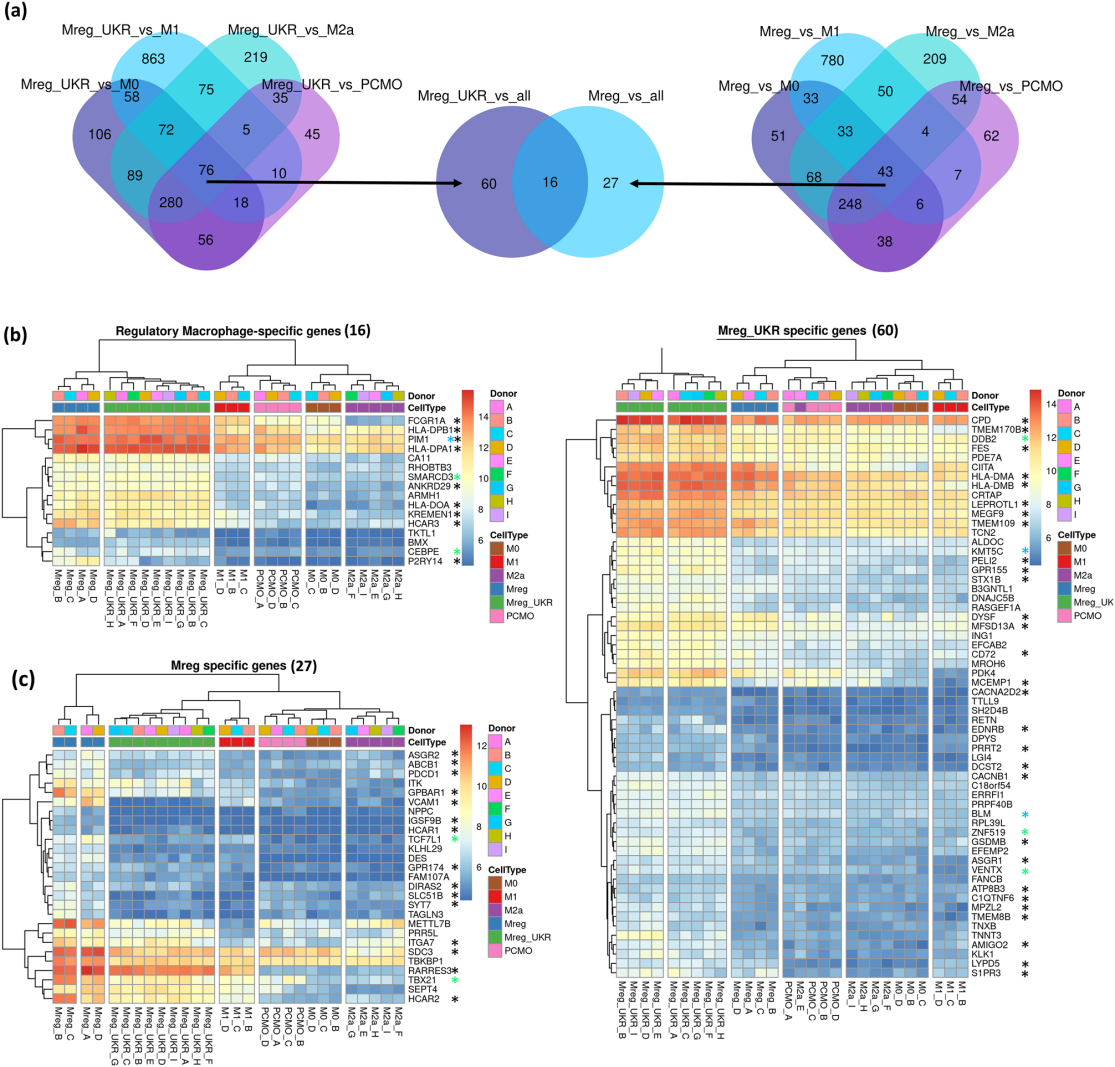
Regulatory macrophage specific genes. (**a**) Stepwise Venn diagram analysis of genes upregulated in Mreg and Mreg_UKR in comparison to other macrophages and PCMO-like cells. Shared gene subset defines regulatory macrophage-specific genes, unique gene subsets define genes specific for each cell type. Heatmap visualization of genes (**b**) common between Mreg and Mreg_UKR; (**c**) specific for Mreg only; (**d**) specific for Mreg_UKR only. Genes encoding membrane-localized proteins (GO:0005886: plasma membrane and/or GO:0016021: integral component of membrane) are marked with black stars. Green stars indicate transcription factors and blue stars—transcription co-factors.

We endeavoured to pinpoint prospective markers for regulatory macrophages from our data. Differential expression gene sets were analysed using Venn diagrams to identify genes specifically upregulated (LFC1) in both Mreg and Mreg_UKR in comparison to M1, M0, M2a and PCMO-like backgrounds. In both regulatory macrophages, 16 genes were commonly upregulated ≥ twofold, with a further 27 genes in Mreg and 60 genes in Mreg_UKR (Fig. 5a). Protein products from the majority of these genes are known to localize to the cell membrane, with specific enrichment for molecules involved in antigen presentation (p < 0.001). Hierarchically clustered heatmap analysis of expression levels for those genes revealed that only a subset was exclusively upregulated in either Mreg or Mreg_UKR (Fig. 5b–d).

Similarly, we defined genes specifically downregulated in regulatory macrophages when compared to other products. Only 4 genes, *FOXP1*, *ZNF827*, *MAOA* and *XYLT1* were identified in Mreg when compared to M1, M2a, M0 and PCMO-like cells. *MAOA* and *XYLT1* belong to the TGFB1 regulatory network and are associated with the M2 phenotype^27,45^. The corresponding list of Mreg_UKR-specific downregulated genes contained 122 protein coding genes. Protein products for the majority of those genes are annotated as being localized to the cell membrane (73 genes to cellular component GO:0016020 membrane), with 15 genes involved in inflammatory response (GO:0006954) and 19 genes involved in cell migration (GO:0016477) (Supplementary Figure S5d). Inflammation associated genes *IL1B*, *CCL3* (MIP-1) and *CCL4* (MIP-1b) were further verified by multiplex ELISA and shown to be secreted at negligible levels in Mreg_UKR (Supplementary Figure S2a). While the regulation of cytokine secretion is a spatiotemporal process, the cytokine panel was nevertheless transformed to log_2_-fold changes and compared to RNA-Seq estimates in order to investigate a possible correlation. Concordance was achieved for a majority of the panel (Supplementary Figure S2b), with only a small subset of cytokines, including IL-1β, *not* secreted proportionally to the number of expressed transcripts (Supplementary Figure S2c).

Based on these data, we have identified a number of genes specific for regulatory macrophages and further distinguishing the two studied regulatory macrophages, namely Mreg and Mreg_UKR.

### Dissecting the differences between regulatory macrophages

**Figure 6:**
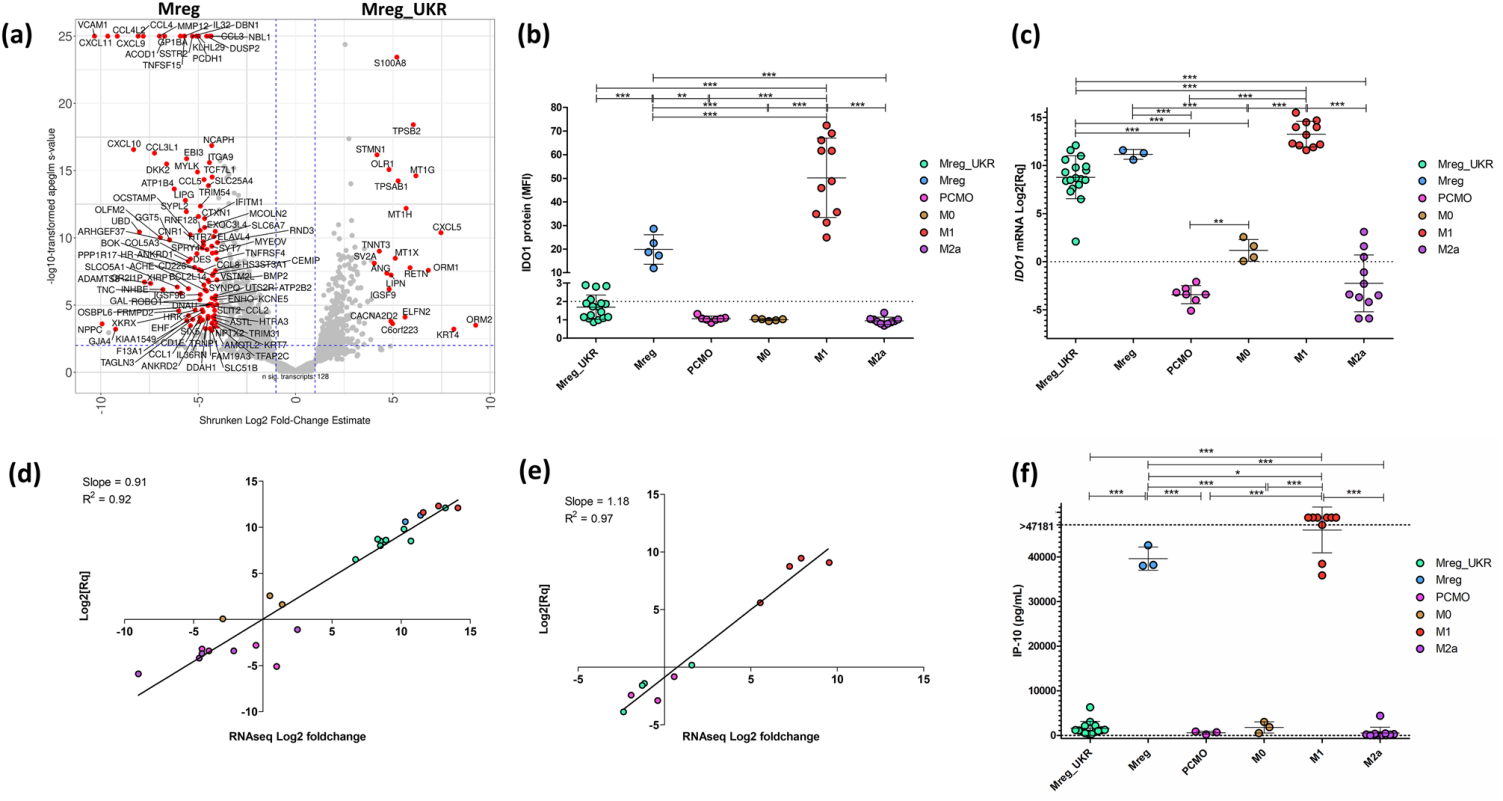
Differences between Mreg and Mreg_UKR exemplified by response to IFN-γ. (**a**) Volcano plot showing differentially expressed genes between the two cell types. Genes increased by ≥ 16-fold are indicated in red. (**b**) IDO1 protein as measured by flow cytometry. **(c)** *IDO1* mRNA as measured by RT-qPCR. (**d**) Expression data obtained by RNA-Seq is concordant with RT-qPCR results for *Ido1* mRNA and (**e**) *IL32* mRNA. (**f**) Secretion levels of IFN-γ-induced IP-10 (*CXCL10*). Data obtained for different donor derived cells is presented as individual points, with mean ± SD for each cell type indicated where appropriate. For panels (**b**), (**c**) and (**f**) significance was calculated using one way ANOVA for each pair-wise comparison with Tukey’s post hoc test in GraphPad Prism and is indicated *p < 0.05, **p < 0.01, ***p < 0.001. Linear regression analysis for data in (**d**) and (**e**) was performed in GraphPad Prism.

As shown above, Mreg and Mreg_UKR differ significantly in expression of a number of cell membrane proteins. Overall transcriptome of the two differs in over 1,000 of genes, with 486 protein coding genes being upregulated in Mreg and 321 protein coding genes in Mreg_UKR, respectively (LFC1) (Fig. 6a; Table 1). Expression of pro-inflammatory chemokines and cytokines, *e.g. CXCL1*, *CXCL9*, *CXCL10* (IP-10), *CXCL11*, *CCL2* (MCP-1), *CCL3* (MIP-1a), *TNF*, *CCL4* (MIP-1b), *CCL5* (RANTES), *CCL8*, *IL32* and *IL1B* was upregulated in Mreg relative to Mreg_UKR. This conclusion was supported by multiplex ELISA measurements for IP-10, MCP-1, MIP-1a and MIP-1b, but not for IL-1β nor TNF-α (Supplementary Figure S2a). Analysis using DAVID of Mreg upregulated genes confirmed enrichment for genes belonging to Cytokine-cytokine receptor interaction, Toll-like receptor, TNF and NF-κB signalling pathways (Table 3). Gene ontology terms for Biological Processes were enriched for GO:0006954:inflammatory response, GO:0002548:monocyte chemotaxis, GO:0007155:cell adhesion, GO:0071356:cellular response to tumor necrosis factor, GO:0070098:chemokine-mediated signalling pathway and GO:0071347:cellular response to interleukin-1 (Table 4). Among these, of special interest was enrichment for GO:00071555:cell adhesion, which contained a number of genes involved in adherens junctions formation and focal adhesion, e.g. *NECTIN4*, *AFDN*, *CD226*, *BCAR1*, *CASS4* and *RND3*. Possibly, this was due to Mreg differentiation as an adherent cell culture on a hard plastic surface, while Mreg_UKR were semi-adherent. Gene ontology terms for cellular components indicated that a majority of those Mreg-specific proteins localized to the membrane or were being secreted (enrichment for GO:0005615:extracellular space, GO:0005886:plasma membrane and GO:0005576:extracellular region) (See Table 4). No specific enrichment was identified by DAVID among the genes upregulated in Mreg_UKR when compared to Mreg apart for elevated expression of metallothionein genes *MT1M*, *MT2A*, *MT1E*, *MT1H*, *MT1X*, *MT1G* shared between the terms GO:0045926:negative regulation of growth and GO:0071294:cellular response to zinc ion (FDR 0.018). Overall, Mreg appear to be more activated and pro-inflammatory than Mreg_UKR. This was corroborated by upstream regulator function prediction in IPA which concluded inhibition of LPS, IFNG, IL1B, TNF as well as NF-κB signalling in Mreg_UKR when compared to Mreg.

**Table 3:** Enriched KEGG pathways (FDR < 0.01) in Mreg upregulated genes (LFC1) when compared to Mreg_UKR.

KEGG pathway	Genes belonging to the term	Enrichment FDR
hsa04060:Cytokine-cytokine receptor interaction	CCL3, TNF, CCL2, TNFRSF12A, CSF1, TNFSF15, CXCL9, CCL8, TNFSF14, TNFRSF8, CCL5, CXCL11, CCL4, CCL7, CXCL10, TNFRSF11A, CXCR5, CCL3L1, IL1B, IL2RG, BMP2, CCL19, CCL4L2, TNFRSF9, INHBE	1.49E−04
hsa04620:Toll-like receptor signalling pathway	CCL3, TNF, MAP2K3, CXCL9, NFKBIA, CCL4L2, MAPK11, CXCL11, CCL5, CCL4, CXCL10, IKBKE, CD80, CCL3L1, IL1B	2.49E−03
hsa04668:TNF signaling pathway	TRAF1, ICAM1, TNF, CCL2, PTGS2, CSF1, MAP2K3, MMP9, NFKBIA, MAPK11, BIRC3, CCL5, CXCL10, VCAM1, IL1B	2.79E−03
hsa04064:NF-κB signalling pathway	TRAF1, ICAM1, TNF, PTGS2, TNFSF14, CCL19, NFKBIA, CCL4L2, BIRC3, CCL4, VCAM1, TNFRSF11A, IL1B	8.69E−03

**Table 4:** Gene Ontology enriched terms (FDR < 0.01) in Mreg upregulated genes (LFC1) when compared to Mreg_UKR.

GO term	Genes belonging to the term	Enrichment FDR
GO:0008009:chemokine activity (MF)	CCL3, CCL2, CCL3L1, CXCL9, CCL8, CCL19, CCL4L2, CCL5, CXCL11, CCL4, CCL7, CXCL10	4.77E−05
GO:0006954:inflammatory response (BP)	CCL3, CCL2, TNF, PTGS2, CSF1, CXCL9, CCL8, TNFRSF8, CCL5, CXCL11, CCL4, CCL7, MMP25, CXCL10, HRH1, PTGIR, TNFRSF11A, CCL3L1, PSTPIP1, IL1B, PTX3, BMP2, DAB2IP, C4B, SPHK1, CHST2, CCL19, CCL4L2, GAL, AIM2, SIGLEC1, TNFRSF9, TNFAIP6, GGT5, ACOD1, HDAC9, PLA2G2D, BMP6	9.27E−09
GO:0002548:monocyte chemotaxis (BP)	CCL3, TNFRSF11A, CCL2, PDGFB, CCL3L1, CCL8, CCL19, CCL4L2, CCL5, CCL4, CCL7	1.63E−04
GO:0007155:cell adhesion (BP)	ACHE, CCL2, TLN2, BCAR1, TNC, PTK7, IL32, PCDHGC3, CCL4, VCAM1, LAMB3, CHST10, SORBS1, ROBO1, COL6A2, PSTPIP1, COL6A1, GP1BA, AFDN, SPON2, BOC, LRFN3, ICAM1, TYRO3, PODXL, SLAMF7, NECTIN4, SLAMF1, TNFAIP6, ITGA9, RND3, LAMA3, CASS4, CD226	2.80E−04
GO:0071356:cellular response to tumor necrosis factor (BP)	ICAM1, DAB2IP, CCL3, CCL2, TRPV1, CCL19, CCL8, CCL4L2, ANKRD1, CCL5, CCL4, CCL7, VCAM1, OCSTAMP, CCL3L1, ACOD1	3.03E−04
GO:0070098:chemokinE−mediated signalling pathway (BP)	CCL3, CCL2, CXCL9, CCL19, CCL8, CCL4L2, CXCL11, CCL5, CCL4, CCL7, CXCL10, CXCR5, CCL3L1	4.83E−04
GO:0071347:cellular response to interleukin-1 (BP)	ICAM1, CCL3, DAB2IP, CCL2, CCL3L1, CCL8, CCL19, CCL4L2, ANKRD1, ACOD1, CCL5, CCL4, CCL7	4.83E−04
GO:0005615:extracellular space (CC)	PDGFB, MMP9, IGFBP6, TNFSF15, TNFSF14, CXADR, CXCL11, C1QC, CXCL10, ACTG1, HIST1H2BK, CCL3L1, SERPINE1, CFH, IL1B, SPON2, EBI3, KLK13, APOO, ICAM1, C4B, CCL4L2, CBR3, GAL, SLIT2, CTSV, TNFRSF9, TNFAIP6, PTGDS, INHBE, F3, SERPINB8, NPPC, ALPL, CCL3, ACHE, CCL2, TNF, ENPP2, TNC, CSF1, CXCL9, CCL8, TIMP4, IL32, NRN1, CCL5, CCL4, CCL7, VCAM1, ZG16B, COL6A2, PTX3, ANGPTL4, BMP2, HIST1H2BC, PODXL, IL1RN, SELENOP, CCL19, IGF1, IL36RN, DKK2, NBL1, SPTBN2, LIPG, AREG, ASIP, CHRD, BMP6	8.96E−06
GO:0005886:plasma membrane (CC)	GPRIN1, SLC9A9, EFNA1, TNFSF15, RRAD, TNFSF14, SYT7, SLC7A5, NRCAM, ACTG1, PTGIR, TRAC, ANK2, ROBO1, SERPINE1, SPRED2, AFDN, SLC51B, MCOLN2, SPRED1, HCAR3, HCAR2, EBI3, DAB2IP, MYO6, C4B, MRGPRF, GPR132, PKD2L1, CD38, SSTR2, GPBAR1, LPAR5, RASGRF1, F3, HTR7, AKAP5, PDGFRA, DSP, SLC38A1, DBN1, CD226, ADD2, COBL, ACHE, IFITM1, ENPP2, TNFRSF12A, NFKBIA, AFAP1L1, NRN1, RAB40B, KCNJ1, P2RY6, CNR1, ADRA2B, LRFN3, S100A16, MYO1B, OSBPL6, PODXL, CPNE6, GAREM1, SPHK1, SYT12, IL1RN, ADGRG6, ABCB1, SLAMF7, S100A14, GPR153, GGT5, ITGA9, LAMP3, CD79A, UTS2R, GPR84, LRRC8A, SLC20A1, TRPV1, SLC16A10, TLN2, MARCKSL1, BCAR1, CXADR, KCNK13, MMP25, SDC3, PCDH1, CTTN, TNFRSF11A, CXCR5, RASL10A, SMAGP, ATP8B1, RHOD, BOC, RHOF, ANO9, SLC22A1, SHC4, ICAM1, RAB39A, MICAL3, MMP15, NECTIN4, RFTN1, SLC9A3R2, PCDHGB5, SLIT2, SIGLEC1, CHRM4, CD80, CEMIP, GRASP, ALPL, TNF, CSF1, FFAR2, XKRX, PCDHGC3, CLEC10A, VCAM1, LINGO1, HRH1, SORBS1, GLIPR1, RASGRP1, LANCL2, LANCL3, PSTPIP1, SLC39A8, IL2RG, CAMK2B, GP1BA, KCNE5, EHD1, YES1, OR2I1P, CBARP, NPR1, IGF1, CD1A, TSPAN15, RGS16, RGS20, TJP1, SLC6A7, P2RY14, GNG10, LRP6, PPP1R13B, CTNS	6.27E−05
GO:0005576:extracellular region (CC)	PDGFB, EFNA1, MMP9, F13A1, IGFBP6, FST, CSPG4, IL4I1, CXADR, CXCL11, C1QC, CXCL10, NRCAM, CCL3L1, SERPINE1, CFH, IL1B, HTRA3, EBI3, KLK13, APOO, C4B, DRAXIN, MMP19, CCL4L2, GAL, SLIT2, CTSV, MMP12, DNASE2B, C1QA, SIGLEC1, C1QB, PTGDS, INHBE, NPPC, CEMIP, PLA2G2D, ADAMTS2, CCL3, ACHE, CCL2, TNF, TNC, CXCL9, C1S, CCL5, CCL4, CCL7, LAMB3, GLIPR1, COL6A2, COL6A1, PTX3, OLFM2, ANGPTL4, BMP2, PLEK, SELENOP, CCL19, IGF1, COL5A3, NTN1, DKK2, PRADC1, LAMA3, VSTM2L, LIPG, LRP6, C11ORF45, BMP6, ENHO	1.86E−03
GO:0030054:cell junction (CC)	ACHE, TRPV1, SYT7, AFAP1L1, NRN1, CXADR, SYP, SMAGP, AFDN, HCAR3, OLFM2, HCAR2, HAP1, LRFN3, SHC4, C4B, SYT12, PSD3, CBARP, IGSF9B, HOMER1, S100A14, FARP1, TJP1, CHRM4, XIRP1, PLEKHA7, SPTBN2, DSP, GRASP, UNC13A	2.13E−03
GO:0005887:integral component of plasma membrane (CC)	GPR84, SLC20A1, TRPV1, SLC16A10, EFNA1, ATP1B4, CSPG4, TNFSF15, KCNK13, CXADR, SLC7A5, GJA5, NRCAM, TSPAN12, PCDH1, PTGIR, TNFRSF11A, TSPAN10, CXCR5, ROBO1, ATP8B1, SMAGP, HCAR3, SLCO5A1, BOC, SLC22A1, ICAM1, TYRO3, SLC25A4, SLC22A23, MRGPRF, NECTIN4, MMP15, TNFRSF9, SSTR2, PODXL2, CHRM4, HTR7, PDGFRA, SLC38A1, CD226, HS3ST3B1, TNF, ENPP2, FFAR2, PTK7, TNFRSF8, ESYT3, HRH1, P2RY6, CNR1, SLC39A8, IL2RG, GP1BA, FUT1, ADRA2B, PODXL, NPR1, CD1A, TSPAN15, SLC6A7, P2RY14, CD79A, UTS2R	5.86E−03

Only the five highest ranked enrichments are presented for each enrichment type.

*MF* molecular function, *BP* biological process, *CC* cellular component types of gene ontology terms.

Response to IFN-γ was decreased in Mreg_UKR, when compared to Mreg and M1 macrophages. This RNA-Seq data conclusion was confirmed by measurement of products of several IFN-γ-induced genes, *IDO1* and *IL32* mRNA by RT-qPCR, IDO1 protein by flow cytometry and CXCL10 (IP-10) cytokine release by multiplex ELISA (Fig. 6b–f). IFN-γ is a potent inducer of transcription of *IDO1*^46,47^. IFN-γ was added during manufacturing of Mreg_UKR, Mreg and M1 macrophages, but not to M2a, M0 or PCMO-like cells. IDO1 was not expressed in M0, M2a and PCMO-like cells on the protein level and was either marginally upregulated (M0) or downregulated (PCMO-like cells) on the level of transcribed mRNA when compared to the CD14 + monocytes (Fig. 6b,c). In M1 and Mreg the IDO1 protein was highly expressed, but was undetectable in several Mreg_UKR batches by flow cytometry. RT-qPCR confirmed the upregulation of *IDO1* (4- to 4,000-fold) in Mreg_UKR (Fig. 6c), which was generally lower than in Mreg and significantly lower than in M1 (p < 0.001). This RT-qPCR data was in agreement with the RNA-Seq data for *IDO1* (Fig. 6d) as well as for another IFN-γ-inducible gene, *IL32*, which was upregulated only in M1 (Fig. 6e). Another IFN-γ-inducible protein IP-10 (*CXCL10*) was measured as a part of a multiplex ELISA. While culture medium of M1 and Mreg contained > 38,000 pg/ml of secreted IP-10, the average secretion from Mreg_UKR cells was < 2,000 pg/ml (Fig. 6f), which further showed that IFN-γ response was attenuated in Mreg_UKR.

### Phagocytic activity of human Mreg macrophages

**Figure 7:**
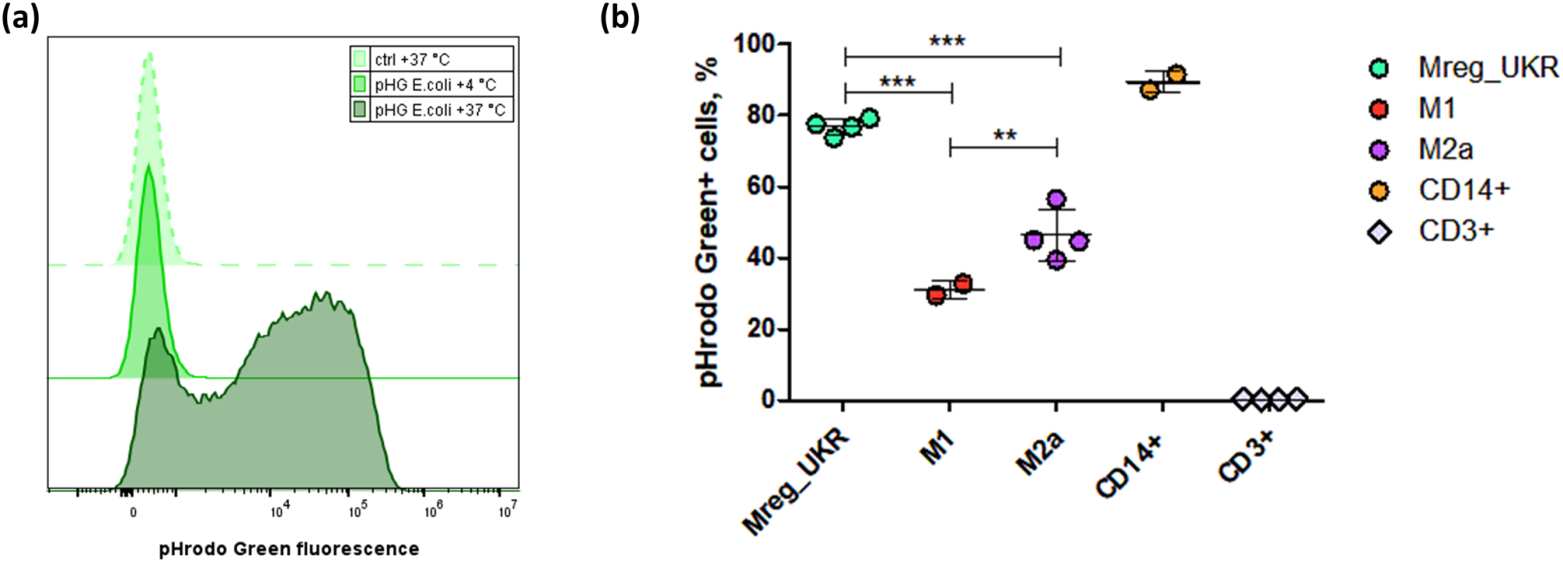
Phagocytosis of pHrodo Green *E. coli* particles by macrophages. Cells were incubated with *E. coli* particles for 1 h and analyzed by flow cytometry. Donor-matched CD14 + monocytes were used as positive controls, while unmatched CD3 + T cells and macrophages with target antigen-coated particles incubated on ice, which precludes active phagocytosis, were used as negative controls. (**a**) Representative histograms of engulfed particles in Mreg_UKR, measured as pHrodo Green fluorescence intensity. The dashed light-green plot indicates the control without particles incubated at + 37 °C. The light-green plot shows the cells with particles co-incubated on ice, and the dark-green plot represents the sample with particles incubated at + 37 °C. (**b**) Dot and whisker plots of the percentages of the cells that had internalized labelled *E. coli* particles. Results are depicted by individual data point percentages of pHrodo Green-positive cells within the live CD45-positive population (live CD45 + CD3 + for T cells) with mean ± SD indicated. Levels of statistical significance of difference in phagocytic ability between the groups was calculated with one way ANOVA with Tukey’s multiple comparison post hoc test and are indicated by **p < 0.01; ***p < 0.001.

Regulatory macrophages had elevated expression of MHC class II molecules according to RNA-Seq data (Fig. 5b) and were predicted to function better in antigen presentation than other types of macrophages. Therefore, we quantitated phagocytosis, which represents an essential step of triggering the innate immune response against bacteria (Fig. 7a). After 1 h 31.2 ± 2.3% of the M1, 46.4 ± 7.2% of the M2a and 76.9 ± 2.3% of the Mreg_UKR macrophages had internalized *E. coli* BioParticles (Fig. 7b). This confirmed that differentially activated macrophages have different phagocytic abilities, with regulatory macrophages being the most active. We had also evaluated phagocytosis of IgG-opsonized particles by Mreg, Mreg_UKR and PCMO-like cells in a preliminary assay. Phagocytic activity of Mreg and PCMO-like cells was even higher than the one of Mreg_UKR (Supplementary Figure S6). While Mreg have previously been reported to be more active in phagocytosis than M1 macrophages^2^, this is the first time (to our knowledge) that increased phagocytosis relative to M2a macrophages was documented.

## Discussion

Cell therapy applications are promising treatments for a variety of diseases. However, success in the fundamental research is rewarded with new challenges while moving towards clinical trials. In cell therapy, the process makes the product and, consequently, the development of GMP-compliant manufacturing and supportive analytics must occur before pre-clinical and clinical studies. An ample analysis toolbox should be established based on an unbiased characterization of the developed product to secure process reproducibility and the resultant product’s potency.

In this study, we sought to deepen the knowledge of regulatory macrophages, their polarization states and the impact of manufacturing on their phenotype. We adapted earlier published manufacturing methods for regulatory macrophages and PCMOs, and assessed their characteristics relative to the comparator macrophages (M0, M1, and M2a). We focused on a set of well documented macrophage markers determined by flow cytometry^1,2,31,48^. However, the produced cell types were not conclusively distinguished using these markers. Furthermore, we wished to understand the consequence of any process change in order to ensure that observed shifts in phenotype do not have an undesired effect on the functionality and/or safety profile of a cell therapy product.

The necessity of an unbiased survey of gene expression for cell therapy products has been emphasized in a study comparing bone marrow stromal cells (BMSCs) produced at different centres according to internal protocols^49^. Hierarchical clustering and principal component analysis of RNA-Seq data showed that variability in manufactured BMSC was greater between different centres than within each centre. This inter-centre discrepancy likely resulted in variation in the BMSCs functions, such as the therapy’s ability to form bone and support haematopoiesis in an in vivo transplant model.

We employed RNA-Seq for comprehensive, unbiased molecular characterization of macrophages included in this study and were able to reliably distinguish all macrophage types. Importantly, RNA-Seq data confirmed that the manufacturing processes were highly reproducible and largely unaffected by donor-to-donor variations. The inflammatory M1 were the most distinct macrophage type that could be also distinguished utilizing few phenotype markers. The non-polarized M0, alternatively activated M2a, and PCMO-like cells had minor differences as visualized in principal component analysis and hierarchical clustering. M-CSF is common in manufacturing of all these cell types and appears to have a greater effect on the cell characteristics than does the source of serum or addition of IL-3 or IL-4. Clinical and in vivo studies suggest that PCMO, much like M2 macrophages, could facilitate tissue remodelling and wound healing^19,50^.

The two regulatory macrophage products clustered apart from each other based on RNA-Seq data analysis. The original regulatory macrophage cell therapy product was developed as an adherent Mreg culture in flasks^30^. Later, the production was up-scaled to a GMP-compliant process (Mreg_UKR), using semi-adherent bag conditions^31^, and in parallel, possibly to minimize handling steps, medium replenishments were abandoned. Macrophages are highly attuned to their microenvironment, and we show here that the putatively minor changes in the regulatory macrophage manufacturing resulted in a clear phenotypic shift which was reflected in surface protein expression, transcriptomics, attenuation of IFN-γ signaling pathways, and activation status reflected in secretome. Considering the plasticity of macrophages it remains to be investigated whether these in vitro differentiated regulatory macrophages will retain their characteristics in a complex in vivo environment.

IFN-γ is a strong activating cytokine which is used to polarize regulatory and M1 macrophages. It activates the expression of number of genes including indoleamine 2,3-dioxygenase (*IDO1*), *IL32* and *CXCL10* (IP-10). However, these genes and corresponding proteins were less expressed in Mreg_UKR than in Mreg, despite receiving an identical IFN-γ dose. Regulatory macrophages exert their immunomodulatory function by transforming naïve CD4 + T cells into anti-inflammatory regulatory T cells^2^ and by suppressing T cell proliferation, both of which are dependent on increased IDO activity^1^. The proposed mechanisms include direct elimination of effector T cells by tryptophan deprivation^1,47^ and indirect immunoregulation via Stat3-mediated induction of regulatory T cells^51^. We observed a clear difference in *IDO1* expression between manufacturing conditions. In the Mreg_UKR, which are produced without medium changes, *IDO1* expression is diminished on both mRNA and protein level. Although Mreg_UKR manufacturing resulted in somewhat “dormant” cells, they retain their propensity for re-activation in a different microenvironment. Our preliminary data suggest that upon stimulation with LPS Mreg_UKR not only upregulate expression of *IDO1,* but also secrete wound healing cytokine IL-10 (unpublished data). This further supports an anti-inflammatory and immunomodulatory role for Mreg_UKR, which in the chronic wound setting could contribute to resolution of inflammation rather than exacerbate the immunopathology.

A recent study demonstrated that regulatory macrophages not only secrete angiogenic proteins, but this secretion increases under induced hypoxia^52^. We did not detect any significant gene expression or secretion of cytokines/growth factors associated with angiogenesis in regulatory macrophages as compared to other phenotypes under basal conditions (i.e. right after harvest). Potentially minor differences in manufacturing or experimental design could account for the observed disagreements.

Determination of objective and measurable biomarkers that would reflect potency are in high demand for potential cell therapies^53^. This study provides a foundation to develop a toolbox for in vitro and in vivo phenotype and potency analyses. Although functional studies were limited to secretion and phagocytosis assays, the pathway and biofunction analysis correctly predicted the known functions of M1 and M2a macrophages. Functional predictions for regulatory macrophages were distinct from those for M1 or M2a and prognosticated Mreg and Mreg_UKR to have anti-inflammatory and antimicrobial attributes. Such properties would be essential in a macrophage cell therapy product that seeks to resolve inflammation in *e.g.* chronic wound, where the reduced capability of macrophages to phagocytose debris and apoptotic neutrophils results in prolonged inflammation^54^. The membrane-localized proteins (identified here by RNA-Seq), especially the ones involved in antigen presentation and phagocytosis, HLA-DPA1, HLA-DPB1, HLA-DOA and CD64 (FCGR1A), are the prime choices for development of novel flow cytometry markers for fundamental research and release panels reflecting both phenotype and therapeutic potency of macrophage products. As part of a release panel, specific cytokines, such as IP-10 for Mreg, could be measured by ELISA directly from the culture medium without sacrificing the cell numbers in the final macrophage product. Another option is to develop a quantitative, multiplex RT-qPCR assay or targeted RNA sequencing panel for rapid analysis of several genes simultaneously. Such a panel could be based on the cell type specific transcripts identified in this study, *e.g.* the ones encoding membrane-localized proteins and specific transcription factors, such as SMARCD3, CEBPE and VENTX for Mreg_UKR. The role of these transcription factors in promoting the regulatory macrophage phenotype has yet to be investigated. Interestingly, VENTX is known to upregulate expression of a number of genes, including CD71, CD64, CD206^55^, which showed increased expression on flow cytometry and/or in RNA-seq. Selection of target transcripts can be further assisted by machine learning based on RNA-Seq data, which, as shown here, is a powerful and operator-independent tool in macrophage classification.

To our knowledge, we provide the first comprehensive analysis of clinically relevant regulatory macrophages and report several unexpected differences. Our transcriptome analysis showed that this method not only distinguishes the different macrophages from each other, but also reveals new potential markers and predicted biofunctions that could be used in the determination of functionality, potency, and ultimately in a product release panel. Process reliability and reproducibility are the cornerstones of successful cell therapy, reducing the risk of changes in cell function which may compromise the safety or efficacy of the final product. We have demonstrated that the manufacturing processes were highly consistent and reproducible. Simultaneously, we show that even seemingly minor process changes affected the transcriptome of the regulatory macrophage product. Further studies could address whether these transcriptomic fluctuations translate into a functionally different cell therapy in vivo*.* Our results emphasize the importance of early process development for translation into clinical settings as well as the importance of concurrent unbiased product characterization to ensure reproducibility of the desired phenotype, functional state, and designation of final release markers for the end product.

## Supplementary information

Supplementary file 1

## Data Availability

RNA-Seq reads, quantified counts, and metadata are available via GEO accession GSE146028 and BioProject PRJNA552427. All other data are available upon request.
