# Supplementary material for: Transcriptomics uncovers substantial variability associated with alterations in manufacturing processes of macrophage cell therapy products
Source: Sci Rep. 2020 Aug 20;10:14049. doi: 10.1038/s41598-020-70967-2 (PMC7441152; doi:10.1038/s41598-020-70967-2)
Supplement: Supplementary file 1 [file 41598_2020_70967_MOESM1_ESM.pdf]

Transcriptomics uncovers substantial variability associated with alterations in manufacturing processes of macrophage cell therapy products

Olga L. Gurvich[1,*], Katja A. Puttonen[1,*], Aubrey Bailey[1], Anssi Kailaanmäki[1], Vita Skirdenko[1], Minna Sivonen[1], Sanna Pietikäinen[1], Nigel R. Parker[2], Seppo Ylä-Herttuala[2], Tuija Kekarainen[1,**]

[1]Kuopio Center for Gene and Cell Therapy, Microkatu 1S, FI-70210 Kuopio, Finland

[2]A.I. Virtanen Institute, University of Eastern Finland, 70211 Kuopio, Finland

*authors contributed equally to this work

**correspondence should be addressed to Tuija Kekarainen; tuija.kekarainen@kct.fi

Supplementary Information.

Supplementary Figures:

Supplementary Figure S1. Phenotype of Macrophages by Flow Cytometry.

Supplementary Figure S2. Cytokine secretion data as measured by multiplex ELISA.

Supplementary Figure S3. Characterization of PCMO.

Supplementary Figure S4. Behaviour of major M2a and M1 specific gene networks in Mreg, Mreg_UKR and PCMO as identified in IPA.

Supplementary Figure S5. Characterization of regulatory macrophages.

Supplementary Figure S6. **Phagocytosis assay with Mreg, Mreg_UKR and PCMO-like cells**

Supplementary Tables:

Supplementary Table S1. Antibody panel for flow cytometry characterization of macrophages.

Supplementary Table S2. Known marker gene sets for M1 (162) and M2a (123) polarized macrophages.

Supplementary Table S3. PCMO markers from literature.

Supplementary Table S4. Top activated regulator networks as identified by Ingenuity Pathway Analyser (IPA) for M2a_vs_M1 upregulated genes (M2a-specific).

Supplementary Table S5. Top activated regulator networks as identified by Ingenuity Pathway Analyser IPA for M1_vs_M2a upregulated genes (M1-specific).

Supplementary Table S6. Markers described in literature for regulatory macrophages

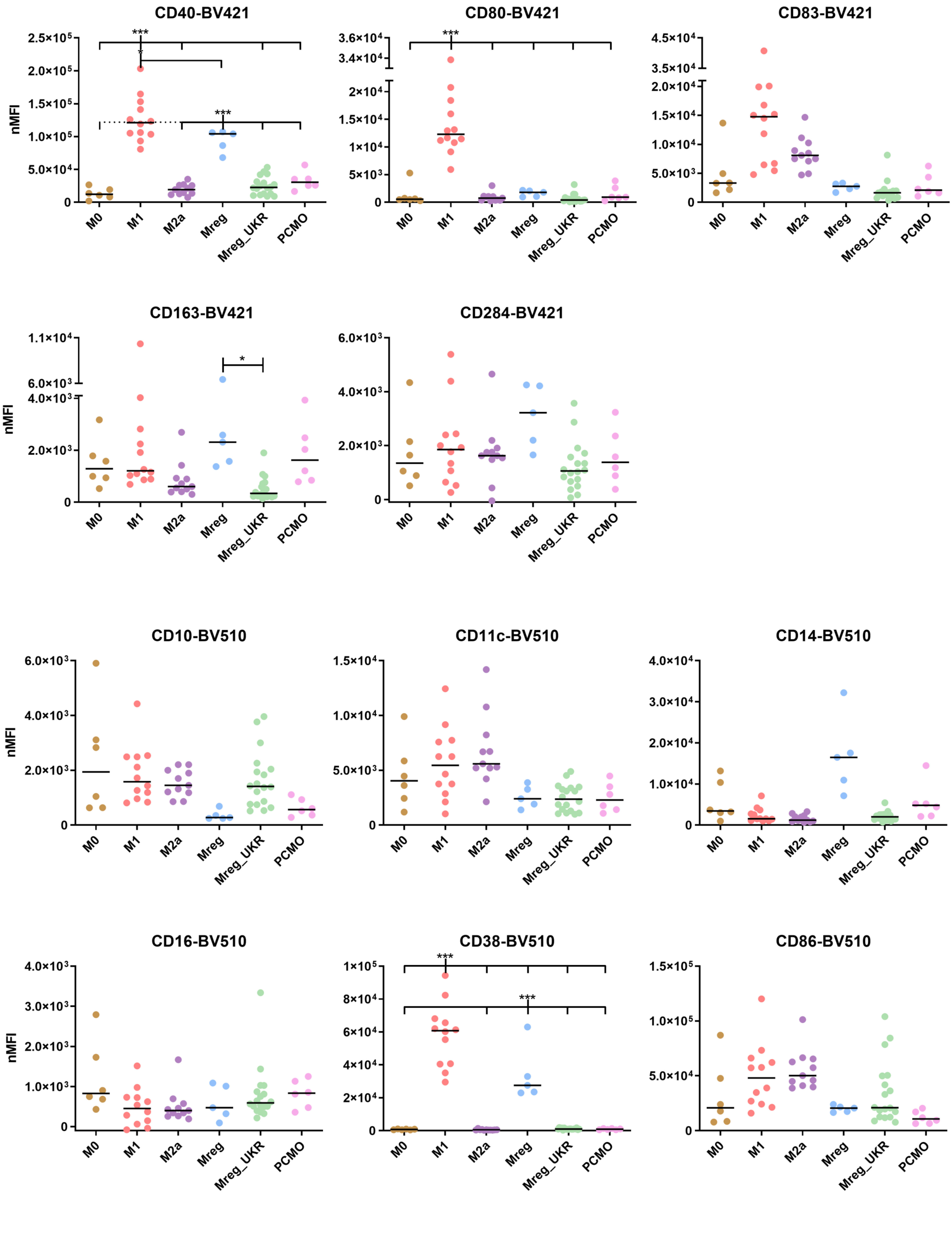

Supplementary Figure S1. Phenotype of Macrophages by Flow Cytometry

The background-subtracted, normalized median fluorescence intensity (nMFI) of 23 extracellular markers employed in the study. p-values are indicated for those cell subsets that have been specifically addressed in the article: * = $p < 0.05$, ** = $p < 0.01$, *** = $p < 0.001$. Significance was calculated using One-way ANOVA with Dunnett post test.

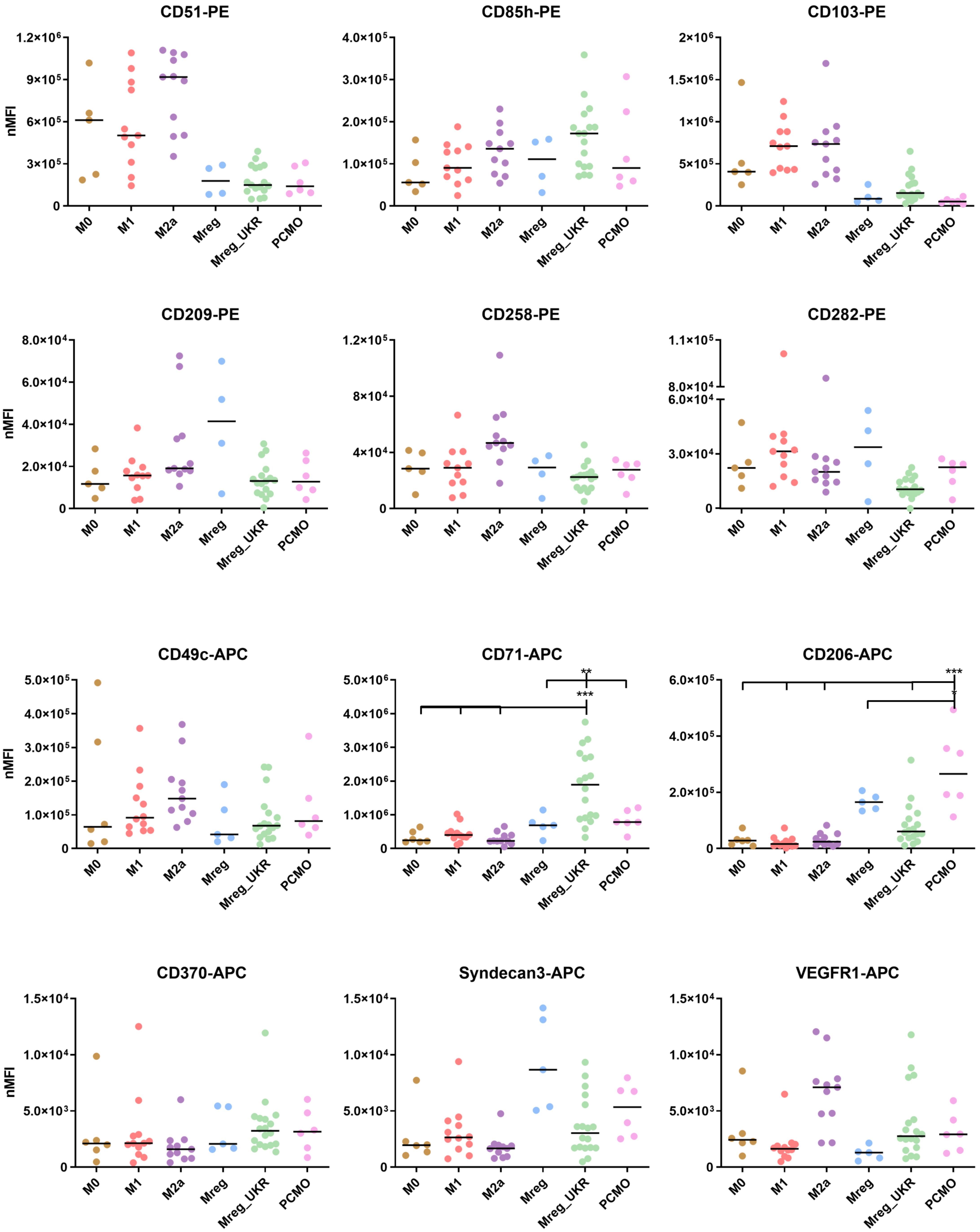

Supplementary Figure S1. Phenotype of Macrophages by Flow Cytometry (continued)
The background-subtracted, normalized median fluorescence intensity (nMFI) of 23 extracellular markers employed in the study. p-values are indicated for those cell subsets that have been specifically addressed in the article. * = $p < 0.05$, ** = $p < 0.01$, *** = $p < 0.001$. Significance was calculated using One-way ANOVA with Dunnett post test.

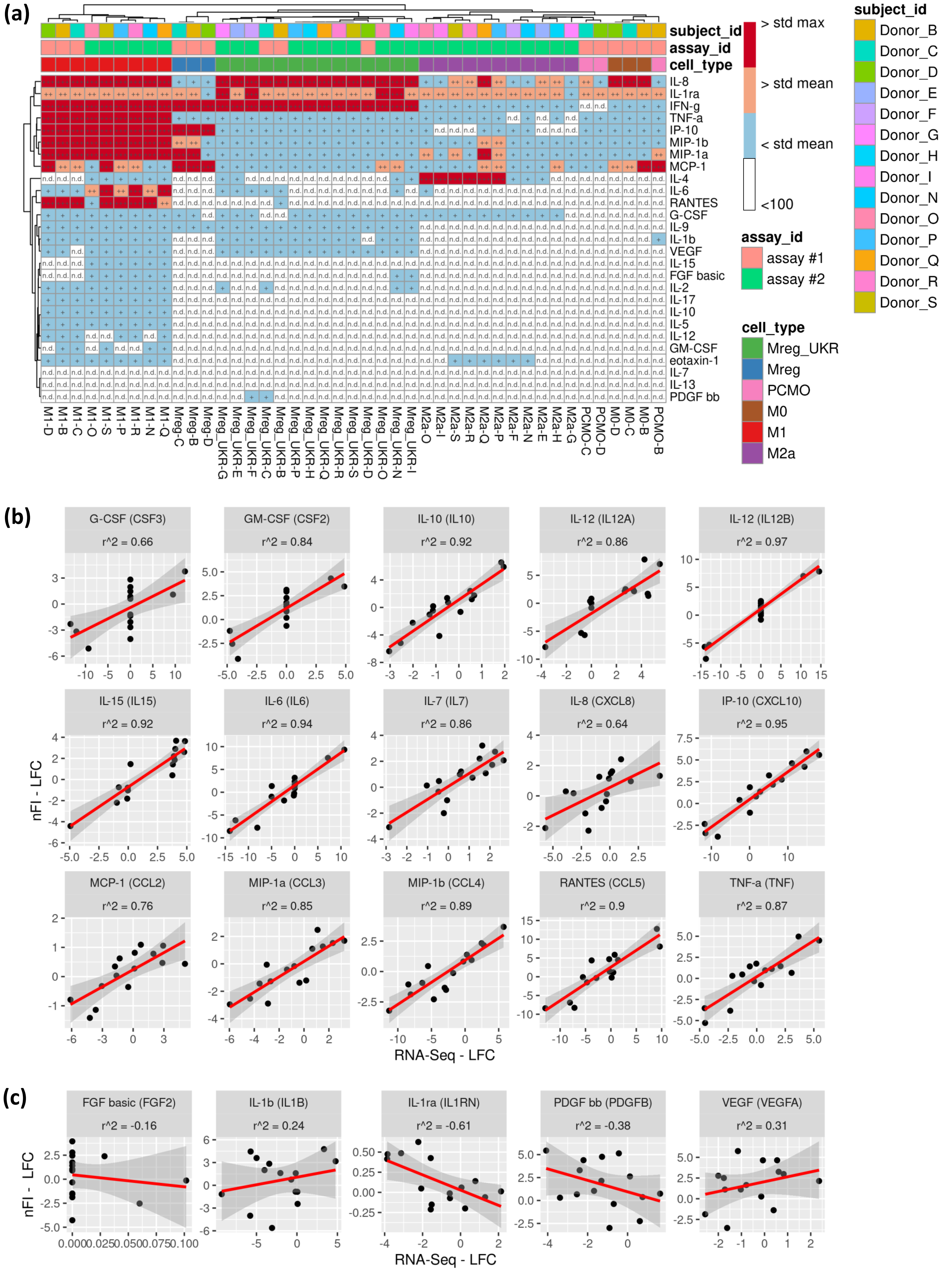

Supplementary Figure S2. Cytokine secretion data as measured by multiplex ELISA. **(a)** A heatmap representing the binned, normalized fluorescence intensities (nFI) represented as the median per cell type, as observed by multiplex ELISA. Values below 100 units were considered to be potentially spurious and as such, their presence was considered to be "not-detected" (n.d.). Values which were within the detectable range, but below the EC50 were considered "present" and indicated with a "+". Similarly, values above the EC50 point, but below the upper asymptote were marked "++". Intensities which were above the manufacturer's highest standard were labelled "+++". Rows and columns clustered by complete linkage clustering. Cytokines, for which level of secretion **(b)** is in concordance and **(c)** is not in concordance with RNA-Seq expression data. Pairwise Log2-fold changes between cell types as observed by RNA-Seq expression (x-axis) and Multiplex ELISA (y-axis), plotted concordantly. Zeros were inserted where values were below established thresholds. Cytokines, which were not expressed on the RNA level from RNA-Seq data, are omitted (IFN-γ, IL-4, IL-13, IL-17A, IL-2, IL-5, IL-9, Eotaxin).

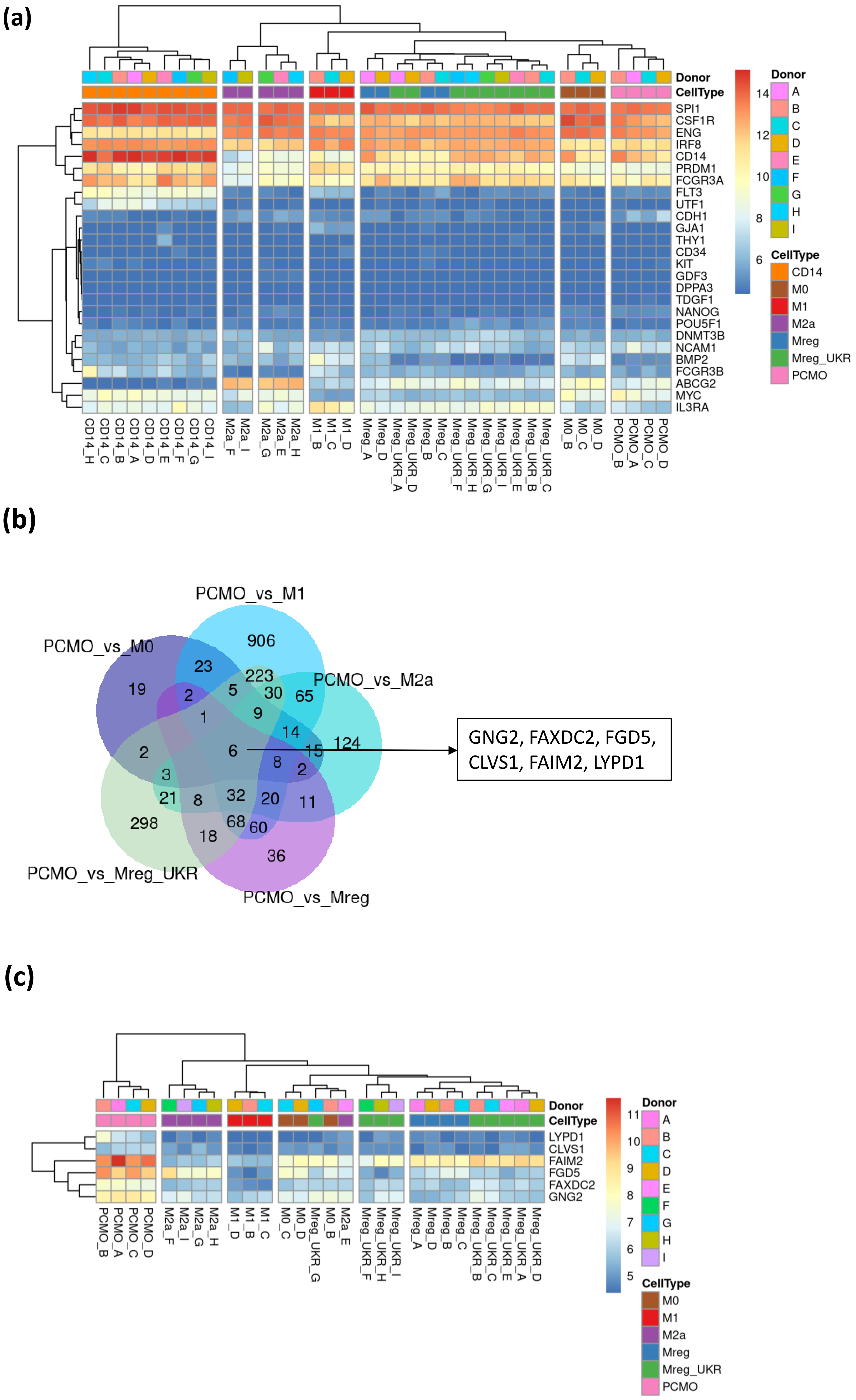

Supplementary Figure S3. Characterization of PCMO-like cells. **(a)** Expression of literature markers for PCMO. **(b)** Venn diagram analysis for identification of PCMO-like markers from our data. Compared are sets of genes upregulated in PCMO-like cells (2-fold, s-value < 0.01) relative to all other macrophages. **(c)** Expression heatmap of the genes defined as intersection in panel (b).

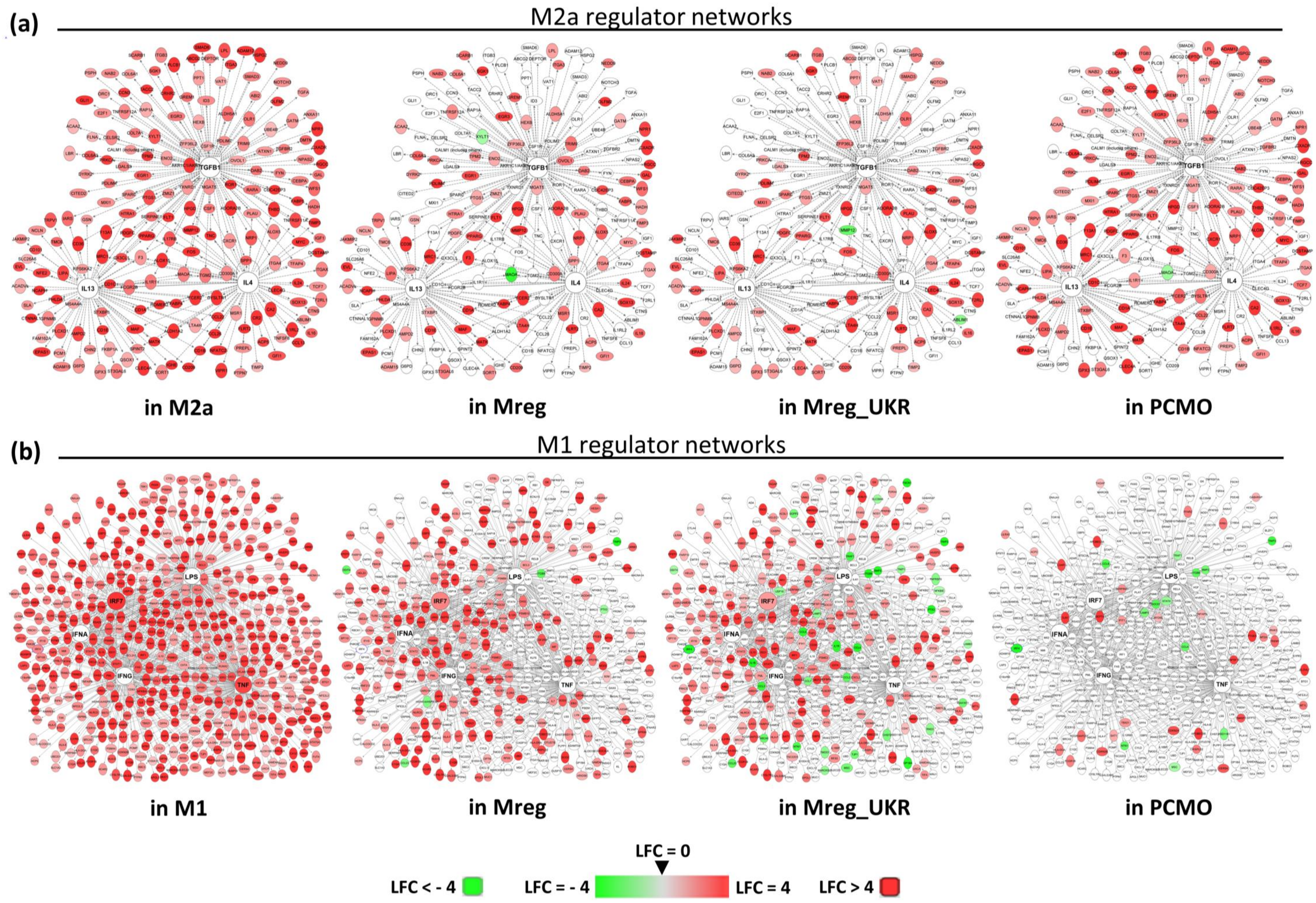

Supplementary Figure S4. Behaviour of major M2a and M1 specific gene networks in Mreg, Mreg_UKR and PCMO as identified in IPA. **(a)** Top 3 regulator networks of M2a__vs__M1 upregulated genes: IL4, IL13 and TGFB1; **(b)** Top 5 regulator networks of M1__vs__M2a upregulated genes: IFNG, LPS, IFNA, IRF7 and TNF; Relative gene expression levels for genes in networks for Mreg, Mreg_UKR and PCMO-like cells is presented relative to M1 for M2a-specific networks and relative to M2a for M1-specific networks.

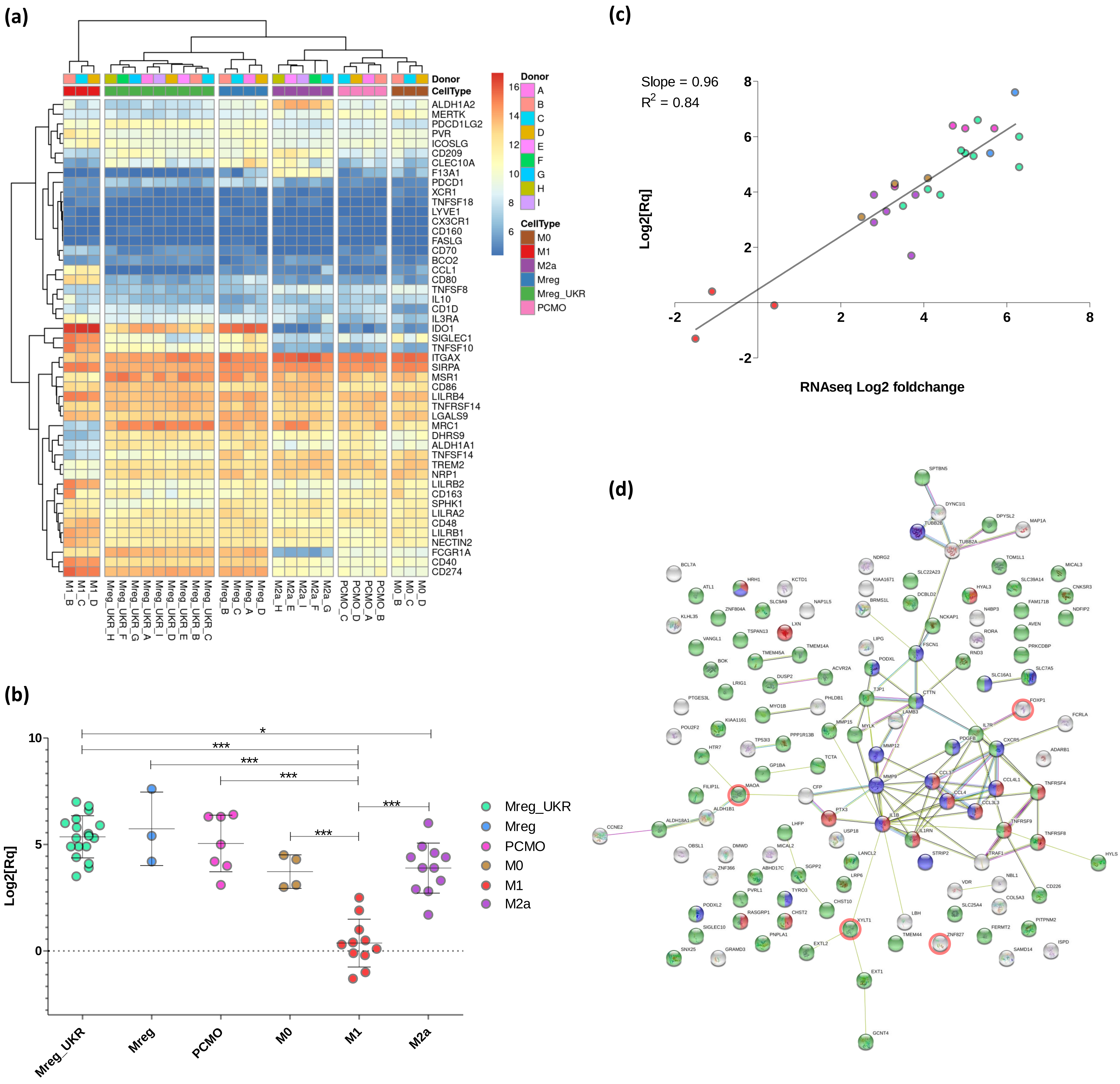

Supplementary Figure S5. **Characterization of regulatory macrophages**. **(a)** Expression heatmaps for regulatory macrophage literature markers; **(b)** RT-qPCR analysis of expression of Mreg marker DHRS9; **(c)** Correlation of RT-qPCR quantitation of *DHRS9* with RNA-Seq data; **(d)** Gene networks identified by string among most down-regulated genes. In green are genes assigned to gene ontology term GO:0016020 membrane, in red – to GO:0006954 inflammatory response, in blue – to GO:0016477 cell migration. For panels (b) significance was calculated using one way ANOVA for each pair-wise comparison with Tukey's post hoc test in GraphPad Prism and is indicated *p < 0.05, **p < 0.01, ***p < 0.001. Linear regression analysis for data in (c) was performed in GraphPad Prism.

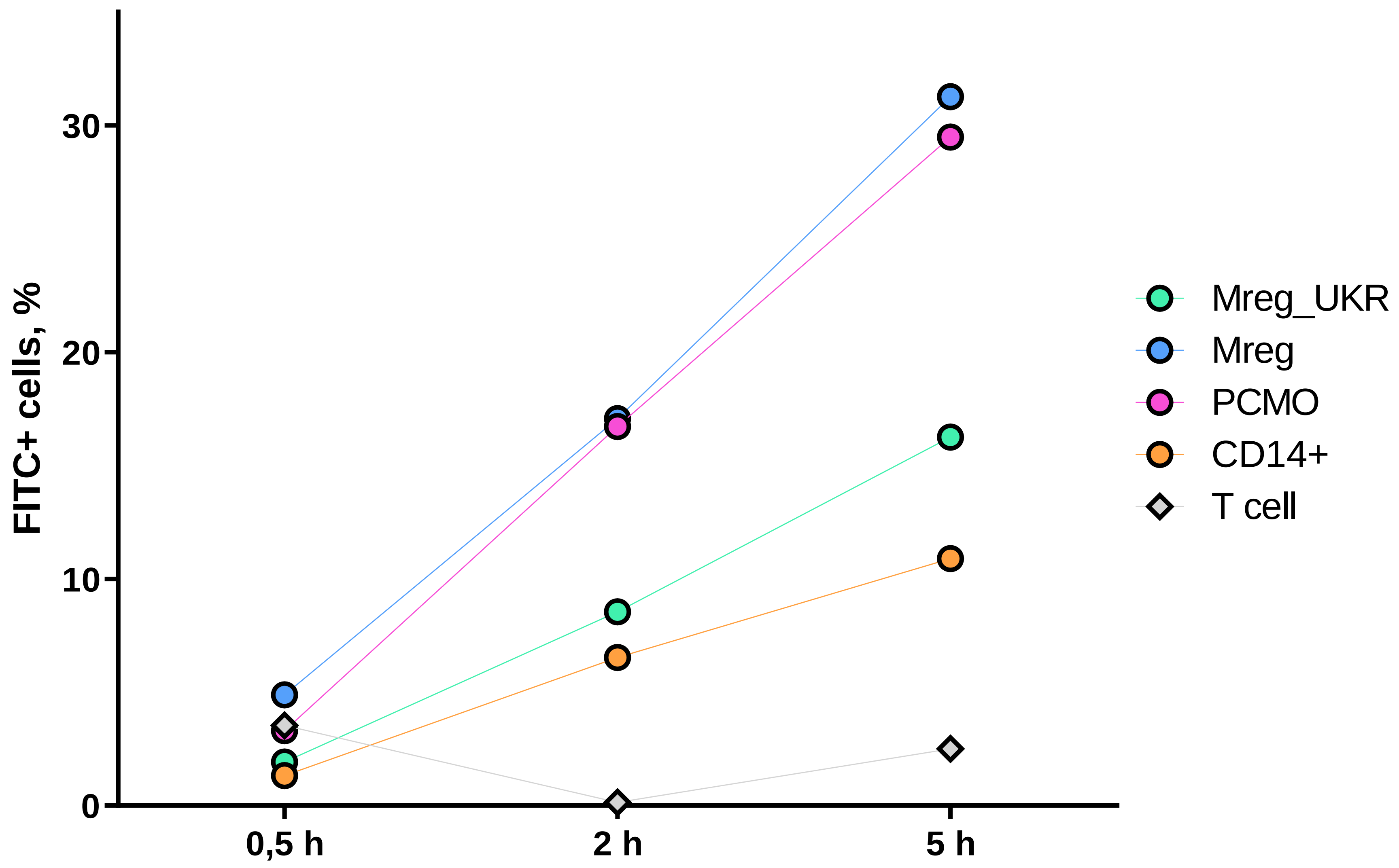

Supplementary Figure S6. **Phagocytosis assay with Mreg, Mreg_UKR and PCMO-like cells**. Assay was performed using Phagocytosis Assay Kit (IgG FITC) (Cayman Chemical, cat. 500290) with latex beads-rabbit IgG-FITC. One million macrophages/monocytes and half a million of T cells were seeded in 1ml of medium and adherent cells were allowed to adhere prior to addition of IgG-FITC beads 200 µl/well. Cells were incubated at +37 °C, 5% CO_2 for 0-5 h and collected at indicated time points. Cells were stained with Sytox-Orange Dead Cell Stain (ThermoFisher S34861) and then resuspended in 90 µl assay buffer. 10 µl of Trypan blue was added to quench surface FITC fluorescence. Phagocytosis was determined as an increase in FITC fluorescence relative to the one observed with controls without beads and calculated as the percentage of FITC-positive cells from Sytox-Orange negative viable population. This assay was performed only once and was later substituted with a quicker and less cumbersome assay presented in the main text.

Supplementary Table S1. Antibody panel for flow cytometry characterization of macrophages. When different from a common name, HGNC names are provided and underlined.

Reaction tube	BV421	BV510	FITC	PE	PE/Cy7	APC
1	CD284<br>TLR4	CD86<br>B7-2	Sytox Gr.	CD209<br>DC-SIGN	CD45<br>PTPRC	CD370<br>Clec9a
2	CD83<br>HB15	CD38<br>cADPrh	Sytox Gr.	CD85h<br>ILT1, LILRA2	CD45<br>PTPRC	CD71<br>TfR1, TFRC
3	CD80<br>B7-1	CD10<br>CALLA MME	Sytox Gr.	CD103<br>ITGAE	CD45<br>PTPRC	Syndecan-3<br>SDC3
4	CD163<br>HbSR	CD14	Sytox Gr.	CD282<br>TLR2	CD45<br>PTPRC	CD206<br>MRC1
5	CD40	CD16<br>FCGR3A	Sytox Gr.	CD51<br>ITGAV	CD45<br>PTPRC	VEGFR1<br>FLT1
6	(CD3)*<br>CD3E	CD11c<br>ITGAX	Sytox Gr.	CD258<br>LIGHT TNFSF14	CD45<br>PTPRC	CD49c<br>ITGA3
7	N/A	N/A	Sytox Gr.	N/A	CD45<br>PTPRC	N/A

BV421: Brilliant Violet 421, BV510: Brilliant Violet 510, FITC: fluorescein isothiocyanate, PE: R-phycoerythrin, Cy7: cyanine 7, APC: allophycocyanin, Sytox Gr.: SYTOX Green dead cell dye (Thermo Fisher Scientific). All antibodies were purchased from Biolegend, except for Syndecan-3 (R&D Systems) and VEGFR1 (Miltenyi Biotech).

*used to assess T cell contamination in the product.

Supplementary Table S2. Known marker gene sets for M1 (162) and M2a (123) polarized macrophages. Cumulative gene set identified through literature[7,20,22,24,35].

**M1 phenotype marker genes**	
Upregulated in comparison to M0 and M2a (132)	AK3, SLC6A12, PSMA2, FAM49A, RELB, DUSP10, PCNX1, STAT3, CLEC4E, UBE2L6, RCN1, TLR2, APOL2, RELA, TYMP, SLC31A2, FCGR1A, SAT1, LRRK2, CLEC4D, GK, HLA-F, WARS, STAT1, GADD45G, IL1B, STX11, PSMB9, OPTN, NFKBIZ, PSME2, APOL6, SLC25A28, FCGR1B, IFI35, PFKFB3, XRN1, OAS3, BIRC3, OAS1, SNX10, IFITM2, SCO2, TAP1, RIPOR2, PARP14, NAMPT, CD40, XAF1, SLC2A6, FAS, SOD2, IFIH1, APOL1, OAS2, SLAMF7, HSD11B1, TNF, IRF7, BATF2, CHI3L2, MX1, IL15, USP18, FPR2, EDN1, LIMK2, VAMP5, IFIT3, ISG15, HERC5, GBP2, IL7R, IRF1, CCL20, INHBA, GUCY1A1, PLAAT4 (RARRES3), OASL, PDE4B, IFIT2, IFI44L, PLA1A, CCL5, APOL3, GBP1, SERPING1, PTGS2, IL15RA, C1S, IL32, IL6, CD80, GCH1, CFB, HESX1, C1R, RSAD2, MUC1, APOBEC3A, CD38, IGFBP4, UBD, GBP5, TNFSF10, CCL15, GBP4, IFI27, IDO2, IL31RA, ISG20, TNFAIP6, IFITM1, LAG3, ETV7, AIM2, SLCO5A1, ANKRD22, LAMP3, CXCL11, CXCL10, HAPLN3, IL2RA, CCR7, IDO1, IL12B, CXCL9, ACOD1, CCL8, CXCL13, SEPTIN4 (SEPT4), CALHM6
Upregulated in comparison to M2a only (8)	GPR84, CLEC6A, PTX3, PAG1, MYD88, IL23A, BCL2A1, RHBDF2
Upregulated in comparison to M0 only (3)	PFKP, VCAM1, CXCL8
No reliable expression change detected (19)	TNFRSF1B, CCL19, SLC7A5, SPHK1, VCAN, PDGFA, ATF3, ADAM28, CYBB, DUSP6, ITGAL, ITGB7, PIM1, SLC22A15, SNTB1, TRIM69, SCIN, IRF3, IFNB1
**M2a phenotype marker genes**	
Upregulated in comparison to M0 and M1 (24)	FCER2, CCL22, CD209, HS3ST2, ALOX15, FGL2, CD1C, CD1E, ESPNL, F13A1, GALNT18, MAOA, PPP1R14A, RAMP1, SPINT2, IL17RB, CD180, ST8SIA6, CHDH, CCL26, CTNNAL1, DNASE1L3, CD226, CD1B
Upregulated in comparison to M1 only (51)	LPAR6, TGFBR2, TLR5, CD302, MSR1, CLEC7A, CLEC10A, MS4A6A, CD36, MS4A4A, MRC1, IGF1, CCL13, SLCO2B1, SLC4A7, SLC38A6, HEXB, LIPA, ADK, CERK, LTA4H, CA2, CHN2, EGR2, MAF, CARD9, CD1A, CDR2L, CMTM8, CRIP1, GATM, GPD1L, KCNK6, PLAU, PTGS1, RGS18, SHPK, ZNF789, GPR34, PTGFRN, THBD, CD200R1, KLHL13, FAXDC2, TMEM71, METTL7A, IL1R1, IL27RA, STAB1, CLEC4A, PPM1L
No reliable expression change detected (48)	P2RY13, HRH1, CXCR4, P2RY14, CCL23, CCL18, CTSC, HNMT, TPST2, HS3ST1, TGFB1, SELENOP, FN1, GAS7, ADAM19, ARRB1, BZW2, CCL17, CLIC2, DUSP22, EMILIN2, FOXQ1, FSCN1, FZD2, GSTP1, ITM2C, MAP4K1, MAPKAPK3, MFNG, NMNAT3, OSBPL7, P2RY11, PALLD, PAQR4, PELP1, PON2, REPS2, RRS1, S100A4, SEC14L5, TGFBI, TMEM97, VCL, TSPAN15, TMEM37, GNG2, GSDMA, CD93

Supplementary Table S3. PCMO markers from literature. When different from common name HGNC designation is provided in brackets.

Marker genes*	Source
CD14, CD90 (THY1), CD115 (CSF1R), CD123 (IL3RA), CD16- (FCGR3A, FCGR3B), CD34-, CD117- (KIT), CD135- (FLT3), PU.1 (SPI1), PRDI-BF/BLIMP-1 (PRDM1), IRF8	Dresske et al 2006[41]
POU5F1, NANOG, MYC, GDF3, DPPA3, ABCG2, connexion-43 (GJA1), NCAM1, DNMT3B, UTF1, BMP2, TDGF1, CDH1, CD105 (ENG)	Ungefroren et al 2016[40]

***** "-" indicates absence of expression according to referenced source.

Supplementary Table S4. Top activated regulator networks as identified by Ingenuity Pathway Analyser (IPA) for M2a_vs_M1 upregulated genes (M2a-specific). Z-scores and p-values are reported by IPA.

Upstream Regulator	Activation z-score	p-value of overlap	Target molecules in dataset
IL13	6.0	5.84E-19	ACADVL,ADAM15,ALDH1A2,ALOX15,AMPD2,CCL22,CCL26,CD101,CD1A, CD1B,CD1C,CD1E,CD209,CD36,CHN2,CLEC4A,CTNNAL1,CX3CL1,CYSLTR1, EPAS1,EVL,F13A1,F3,FABP4,IL17RB,FAM162A,FCER2,FCGR2B,FKBP1A, G6PD,GPNMB,GPX3,GSN,HOMER2,HTRA1,IARS,IGHE,IL1R1,JAKMIP2, LIPA,LTA4H,MAF,MAOA,MATK,MRC1,MS4A4A,NCAPH,NCLN,NFE2,PCM1, PDGFC,PHLDA1,PLCXD1,PPARG,QSOX1,RPS6KA2,SERPINE1,SLA,SLC26A6, SORT1,SPINT2, ST3GAL6,STXBP1,TGM2,TMC6,TRPV1
IL4	5.8	4.66E-09	ABLIM1,ACP5,ADORA2B,ALDH1A2,ALOX15,ALOX5,CA2,CCL13,CCL22, CCL26,CD1A,CD1B,CD1C,CD209,CD300A,CLEC4G,CR2,CSF1,CTNS,CX3CL1, CXCR1,CYSLTR1,DCSTAMP,F2RL1,F3,FABP4,FCER2,FCGR2B,FLRT2,FLT1,FOS, GFI1,HOMER2,HPGD,IGF1,IGHE,IL16,IL17RB,IL1R1,IL1RL2,IL24,ITGA4, ITGAX,LTA4H,MAF,MAOA,MATK,MMP12,MRC1,MSR1,MYC,NFATC2,NRP1, PLAU,PPARG,PREPL,PTPN7,SERPINE1,SOX13,SPINT2,SPP1,TCF7,TFAP4, TGM2,THBD,TIMP2, TIMP3,TNC,TNFRSF11A,TNFSF8,VIPR1
TGFB1	5.3	8.91E-09	ABCG2,ABI2,ACAA2,ADAM12,ADORA2B,AKR1C1/AKR1C2,ALDH5A1, ALOX15,ALOX5,ANXA11,ATP13A3,ATXN1,CALM1,CCN3,CD300A,CD36, CDC42EP3,CEBPA,CELSR2,CITED2,COL6A1,COL6A3,COL7A1,CRHR2,CSF1, CSF1R,CX3CL1,CXADR,CXCR1,DAB2,DEPTOR,DMTN,DYRK2,E2F1,EGR1, EGR3,ENO2,F13A1,F3,FABP5,FAM110B,FLNA,FLT1,FOS,FYN,GAL,GATM, GLI1,GREM1,GSN,HADH,HEXB,HPGD,HSPG2,HTRA1,IARS,ID3,IGF1,IL17RB, IL1R1,ITGA3,ITGA4,ITGB3,LBR,LGALS3,LPL,MAOA,MGAT5,MMP12,MRC1, MXI1,MYC,NAB2,NEDD9,NOTCH3,NPAS2,NPR1,NRP1,OLFM2,OLR1,ORC1, OVOL1,PDGFC,PDLIM4,PDLIM7,PLAU,PLCB1,PPARG,PPT1,PRKCA,PSPH, PTGS1,RAP1A,RARA,RGCC,ROR1,SCARB1,SERPINE1,SGK1,SMAD3,SMAD6, SPARC,SPP1,TACC2,TFAP4,TGFA,TGFBR2,TGM2,THBD,TIMP3,TNC, TNFRSF11A,TNFRSF12A,TPM2,TRIM9,TXNRD1,UBE4B,VAT1,WFS1,XYLT1, ZFP36L2,ZMIZ1,ZNF365

Supplementary Table S5. Top activated regulator networks as identified by Ingenuity Pathway Analyser IPA for M1_vs_M2a upregulated genes (M1-specific). Z-scores and p-values are reported by IPA.

Upstream Regulator	Activation z-score	p-value of overlap	Target molecules in dataset
lipopolysaccharide (LPS)	14.8	5.32E-100	ACSL1,ADA,ADAR,ADM,ADORA2A,AGTR1,AMPD3,ANK2,ANKRD22,ANXA3,ANXA7,APOBEC3A,APOBEC3F,APOBEC3G,APOL6,ASS1,BATF,BCL2A1,BCL3,BIRC3,BLZF1,BMP2,C3AR1,CACNA1A,CASP1,CASP4,CASP7,CCDC170,CCL1,CCL2,CCL20,CCL3,CCL4,CCL5,CCL7,CCL8,CCR5,CCR7,CD274,CD38,CD40,CD44,CD48,CD80,CD83,CEACAM1,CFB,CFLAR,CIITA,CLEC4E,CMPK2,COL1A1,CR1L,CREM,CSF2RB,CSF3,CTSL,CXCL1,CXCL10,CXCL11,CXCL13,CXCL2,CXCL3,CXCL9,CYB5A,CYP27B1,CYP2E1,DDX58,DHX58,DLL4,DRAM1,DUSP5,EBI3,RUNX2,EDN1,EIF2AK2,ENPP2,EREG,ETS2,ETV7,FAS,FFAR2,FLOT2,FPR1,FPR2,FSCN1,G0S2,GABARAP,GADD45B,GBP1,GBP5,GCH1,GIMAP7,GJA1,GK,GNA15,HERC5,HESX1,HGF,HIF1A,HIVEP1,HIVEP2,HLA-A,ICAM1,PSMA6,ICOSLG,IDO1,IDO2,IER3,IFI35,IFI44,IFI44L,IFI6,IFIH1,IFIT1,IFIT2,IFIT3,IFIT5,IFITM1,IFITM2,IGFBP4,IL10,PLD1,IL10RA,IL12A,IL12B,IL15,IL15RA,IL18,IL1A,IL1B,IL23A,IL27,IL2RA,IL32,IL4I1,IL6,IL7,IL7R,INHBA,IRAK3,IRF1,IRF2,IRF7,IRF9,ISG15,ISG20,ITGB8,JAK3,KCNE5,KCNJ15,KLF6,KYNU,LAMP3,LIMK2,LITAF,LMNB1,LRRK2,MAP3K8,MARCKS,MCL1,MEFV,MICA,MIF,MMP14,MT2A,MTF1,MX1,MX2,MXD1,MYD88,OAS3,PLSCR1,PMAIP1,NAMPT,NCF1,NFKB1,NFKB2,NFKBIA,NFKBIE,NFKBIZ,NGFR,NOD1,NOD2,NR4A2,NT5C3A,OAS1,OAS2,OASL,OSM,P2RX4,P2RY6,PAX5,PDCD1LG2,PDE4B,PDIA3,PELI1,PFKFB3,PI3,PIM2,PKIG,PLAGL2,PLAT,PML,PRDM1,PSMB10,PSMB8,PSMB9,PSME1,PSME2,PTAFR,PTGES,PTGS2,PTX3,RARRES3,RB1,RELA,RELB,RSAD2,S100A12,S100A8,S100A9,SAA1,SAA2,SAP18,SARM1,SECTM1,SEPT4,SERPINA1,SERPINB1,SERPINB9,SGPP2,SIGLEC1,SLAMF1,SLAMF7,SLC39A8,SOCS1,SOCS2,SOCS3,SOD2,SPTLC2,SSTR2,STAP1,STAT1,STAT2,STAT3,TAGAP,TANK,TAP1,TAPBP,TBK1,TFPI2,TLR2,TLR3,TLR7,TMSB10/TMSB4X,TNF,TNFAIP2,TNFAIP3,TNFAIP6,TNFRSF1A,TNFRSF9,TNFSF10,TNFSF13B,TNFSF15,TNFSF9,STAT4,STEAP4,TNIP1,TNIP3,TRAF1,TRAF2,TREM1,TRIL, TXN,TYMP,USP18,VEGFC,VNN3,WTAP, XAF1,ZFP36
IFNA	9.6	2.99E-98	ACP2,ADAM19,ADAR,AIM2,APOBEC3A,APOBEC3F,APOBEC3G,AUTS2,BCL2A1,BCL3,BST2,C19orf66,C3AR1,CASP1,CCL2,CCL3,CCL5,CCL7,CCR5,CCR7,CD38,CD40,CD70,CD80,CD83,CEACAM1,CHMP5,CIITA,CMTR1,CXCL10,CXCL11,CXCL9,DDIT4,DDX58,DEFB1,DHX58,DYNLT1,EIF2AK2,ENPP2,EPSTI1,FAS,FBXO6,FCGR1A,GBP1,GIMAP4,GNA15,GVINP1,HELZ2,HERC5,HERC6,HLA-F,HLA-L,HSPA1A/HSPA1B,ICAM1,IDO1,IFI16,IFI27,IFI35,IFI44,IFI44L,IFI6,IFIH1,IFIT1,IFIT2,IFIT3,IFITM1,IFITM2,IFITM3,IGFBP4,IL10,IL10RA,IL12B,PARP9,IL12RB1,IL15,IL15RA,IL1B,IL27,IL2RA,IL6,IL7R,IRF1,IRF4,IRF7,IRF9,ISG15,ISG20,LAIR2,LAMP3,LAP3,LILRB4,MCL1,MEFV,MX1,MX2,MYD88,NFE2L3,NFKBIA,NMI,NT5C3A,OAS1,OAS2,OAS3,PARP10,PARP12,PARP14,PHF11,PI4K2B,PIM2,PLAT,PLSCR1,PMAIP1,PML,PPM1K,PRKCE,PSMB9,PTGS2,RARRES3,RBCK1,RELA,RIPK1,RNF213,RSAD2,RTP4,SAMD9,SAMD9L,SAT1,SEPT4,SERPINB1,SERPINB9,SOCS1,SOCS2,SOCS3,SP100,ZBP1,SP110,STAP1,STAT1,STAT2,STAT4,TAP1,TAP2,TBX21,TDRD7,TLR1,TLR2,TLR3,TLR7,TLR8,TMEM140,TNF,TNFSF10,TNFSF13B,TRANK1,TRIL,TRIM21,TRIM22,TRIM5,TYMP,UBE2L6,UNC93B1,USP18,USP6NL,WARS
IFNG	12.9	4.50E-91	ABCA6,ADM,ADORA2A,AIM2,APOBEC3G,APOL1,APOL6,ASS1,AUTS2,BBC3,BCL2A1,BRCA2,BST2,BTN3A1,C1QA,C1QB,C1QC,CALCOCO2,CASP1,CASP10,CASP7,CCL1,CCL2,CCL20,CCL23,CCL3,CCL4,CCL5,CCL7,CCL8,CCNA1,CCR5,CD274,CD38,CD40,CD44,CD80,CD83,CDKN2A,CDKN2B,CEACAM1,CFLAR,CHI3L2,CIITA,CLEC2D,CLEC4E,COL1A1,CREM,CSF2RB,CSF3,CXCL1,CXCL10,CXCL11,CXCL12,CXCL2,CXCL3,CXCL9,CYLD,CYSLTR2,DDX58,DEFB1,DEPP1,DPP4,EBI3,EDN1,EIF2AK2,ETV7,FAS,FCER1G,FCGR1A,FCGR1B,IFIH1,IFI44L,FCGR3A/FCGR3B,FPR2,GART,GBP1,GBP2,GCH1,GSDMD,HCAR2,HCP5,HERC6,HIF1A,HLA-A,HLA-B,HLA-C,HLA-DOA,HLA-DRA,HLA-E,HLA-F,HLA-G,ICAM1,IDO1,IER3,IFI16,ICOSLG/LOC102723996,IFI27,IFI35,IFI44,IFI6,IFIT1,IFIT2,IFIT3,IFIT5,IFITM1,IFITM3,IGFBP4,IL10,IL10RA,IL12A,IL12B,IL12RB1,IL15,IL15RA,IL18,IL27,IL18BP,IL1A,IL1B,IL23A,IL2RA,IL31RA,IL32,IL4I1,IL6,IL7,IL7R,INHBA,IRAK2,IRF1,IRF2,IRF7,IRF9,ISG15,ISG20,JAK3,KLF6,LAG3,LAMP3,LGALS3BP,MAP3K8,MARCKSL1,MEFV,MICA,MIF,MUC1,MX1,MX2,MYD88,WARS,NAMPT,NFE2L3,NFKB1,NFKBIA,NLRC5,NMI,NTN1,OAS1,OAS2,OAS3,OASL,OPTN,OSM,PDCD1LG2,PELI1,PMAIP1,PML,POMP,PSMA4,PSMB10,PSMB8,PSMB9,PSME1,PSME2,PTGS2,PTPN1,RARRES1,RARRES3,RFX5,RIPK1,RIPK2,RSAD2,RTP4,RUNX2,S100A10,S100A8,S100A9,SAMD9,SEPT4,SERPINA1,SERPING1,SERPINH1,SLC1A3,SOCS1,SOCS2,SOCS3,SOD2,SP100,STAT1,STAT2,STAT4,STX11,TAP1,TAP2,TAPBP,TBX21,THEMIS2,TJP1,TLR1,TLR2,TLR3,TLR7,TLR8,TMOD1,TNF,TNFAIP2,TNFAIP6,TNFSF10,TNFSF13B,TNFSF15,TRIL,TRIM21,TRIM22,TSC22D3,TXK,TYMP,UBD,UBE2D1,UBE2L6,USP18,USP6NL,VEGFC
TNF*	12.9	9.59E-73	ADAMTS4,ADM,ADORA2A,AGTR1,AKR1B1,ALOX15B,APOBEC3B,APOL1,ARHGAP23,ARID5B,B2M,CASP1,B4GALT1,BBC3,BCL2A1,BCL3,BIRC3,BMP2,BPGM,BST2,BTG1,BTG3,BTN3A3,C1QTNF1,CA12,CARD16,CASP10,CASP7,CCL2,CCL20,CCL3,CCL4,CCL5,CCL7,CCR5,CCR7,CD274,CD38,CD40,CD44,CD47,CD70,CD80,CD83,CDKN2A,CERS4,CFB,CFLAR,CHI3L2,CHST2,CIITA,CLEC2D,CLEC4E,COL1A1,CRISPLD2,CSF2RB,CSF3,CXCL1,CXCL10,CXCL11,CXCL12,CXCL13,CXCL2,CXCL3,CXCL9,CYLD,CYP27B1,DAXX,DEPP1,DLL4,DPP4,EBI3,DUSP5,DUSP10,EDN1,EHD1,ELOVL7,ENPP2,EXOC3L4,FAS,FUT4,G0S2,GADD45B,GBP1,GBP1P1,GBP2,GBP4,GCH1,GFPT2,GJA1,GNA15,GP1BA,GPD2,GPR84,GSDMD,HERC5,HGF,HIF1A,HIVEP1,HLA-B,HLA-DRA,HLA-F,HLA-J,HSD11B1,HSPA1A/HSPA1B,ICAM1,ICOSLG/LOC102723996,IDO1,IER3,IFI27,IFI6,IFIH1,IFIT1,IFIT3,IFIT5,IFNAR2,IGFBP4,IL10,IL10RA,IL12A,IL12B,IL12RB1,IL15,IL15RA,IL17RD,IL18,IL18BP,IL1A,IL1B,IL23A,IL2RA,IL32,IL4I1,IL6,IL7,IL7R,INHBA,INSIG1,IRAK2,IRAK3,IRF1,IRF7,ISG15,ITGA10,ITGB8,KCNJ2,NCF1,NAMPT,KIAA1671,KIF3C,KL,KLF6,KYNU,L3MBTL3,LAMA3,LAMB3,LAMC2,LAMP3,LITAF,LSS,LYN,ZFP36,MARCKSL1,MCL1,MEF2C,MEFV,MICA,MIF,MMP14,MSC,MT1A,MT1L,MT2A,MUC1,MYD88,NCK1,NET1,NFE2L2,NFKB1,NFKB2,NFKBIA,NFKBIE,NFKBIZ,NID1,NINJ1,NKX3-1,NOD2,NOTCH4,NR4A2,NTN1,OAS1,OAS2,OASL,OPTN,OSM,OSMR,WTAP,OTUD7B,P2RY6,P3H2,PARP14,PDZD2,PI3,PIM2,PLA1A,PLA2G4C,PLAGL2,PLAT,PLPP1,PLSCR1,PLVAP,PMAIP1,PML,PRDM1,PRRG4,PRSS23,PSEN1,PSMB10,PSMB8,PSMB9,PSME1,PSME2,PTGES,PTGS2,PTX3,RARRES3,RELA,RELB,RFTN1,RFX5,RIPK2,RND1,RND3,ROBO1,RUNX2,S100A8,S100A9,S1PR3,SAA1,SAMD4A,SAMD9,SAT1,SCN9A,SCO2,SERPINB1,SERPINB8,SERPINB9,SLC1A2,SMURF1,SOCS1,SOCS2,SOCS3,SOD2,ST8SIA4,STAT1,STAT4,STAT5A,SYNGR3,TAP1,TAPBP,TBX21,TCHH,TDRD7,TFPI2,TIFA,TJP1,TLR2,TLR3,TM4SF1,TNF,TNFAIP2,TNFAIP3,TNFAIP6,TNFAIP8,TNFRSF9,TNFSF10,TNFSF13B,TNFSF15,TNFSF9,TNIP1,TRADD,TRAF1,TRAF2,TREM1,TSC22D3,TYMP,UACA,UBD,UBQLN2,VEGFC
IRF7*	8.9	1.18E-64	ADAR,APOBEC3G,CARD16,CASP4,CCL5,CCL8,CCNA1,CD40,CD80,CTLA4,CXCL10,CXCL9,DDX58,DHX58,DNAJA1,GBP1,GBP3,GBP4,GBP5,HERC5,IDO1,IFI16,IFI35,IFI44,IFI44L,IFI6,IFIH1,IFIT1,IFIT2,IFIT3,IFITM1,IFITM2,IFITM3,IL12A,IL15,IL15RA,IL27,IL4I1,IRF1,IRF9,ISG15,ISG20,JAK2,LILRA5,MAP3K8,MCL1,MICB,MX1,MX2,NAMPT,NMI,OAS1,OAS2,OAS3,OASL,PELI1,PMAIP1,PSMB10,PSMB8,PSMB9,PSME1,PSME2,RIPK2,RSAD2,RTP4,S100A8,SOCS1,STAT1,STAT2,TAP1,TAP2,TLR8,TNFAIP8,TNFSF10,TNFSF13B,TOR1B,TRAF1, TRIM21,TRIM22,TRIM5,UBE2L6,USP18,XAF1,ZBP1

*TNF and IRF7 expression was upregulated in M1_vs_M2a 3.7 and 5.2 LFC respectively

Supplementary Table S6. Markers described in literature for regulatory macrophages. When different from common name HGNC designation is provided in brackets.

Marker genes*	Source
IL10, SPHK1, TNFSF14 (LIGHT or CD258), CCL1	Mosser and Edwards 2008 [35]
IDO1, CD85h (LILRA2), CD86	Hutchinson *et al* 2017 [28]
DHRS9, ALDH1A1, ALDH1A2, BCO2, CD1D	Riquelme *et al* 2017[42]
CX3CR1, CD11c (ITGAX), XCR1, CD172a (SIRPA), CD123 (IL3RA), CD304 (NRP1), CD64 (FCGR1A), CD163, CD169 (SIGLEC1), CD204 (MSR1), CD206 (MRC1), CD209, CD301 (CLEC10A), LYVE1, F13A1, MERTK, ILT2 (LILRB1), ILT3 (LILRB4), ILT4 (LILRB2) TREM2, CD80−, CD273 (PDCD1LG2), CD274, CD275- (ICOSLG), CD279 (PDCD1), CD270 (TNFRSF14), CD112 (NECTIN2), CD155 (PVR), Galectin-9 (LGALS9), CD48, CD70−, GITRL (TNFSF18), CD153 (TNFSF8), CD160−, CD178- (FASLG), CD40, CD253- (TNFSF10)	Riquelme et al 2018[43]

*****"-" indicates absence of expression according to referenced source.
